# DISSECT: deep semi-supervised consistency regularization for accurate cell type fraction and gene expression estimation

**DOI:** 10.1186/s13059-024-03251-5

**Published:** 2024-04-30

**Authors:** Robin Khatri, Pierre Machart, Stefan Bonn

**Affiliations:** Institute of Medical Systems Biology, Center for Molecular Neurobiology, Center for Biomedical AI, University Medical Center Hamburg-Eppendorf, Hamburg, Germany

**Keywords:** Cell deconvolution, Semi-supervised learning, Deep learning

## Abstract

Cell deconvolution is the estimation of cell type fractions and cell type-specific gene expression from mixed data. An unmet challenge in cell deconvolution is the scarcity of realistic training data and the domain shift often observed in synthetic training data. Here, we show that two novel deep neural networks with simultaneous consistency regularization of the target and training domains significantly improve deconvolution performance. Our algorithm, DISSECT, outperforms competing algorithms in cell fraction and gene expression estimation by up to 14 percentage points. DISSECT can be easily adapted to other biomedical data types, as exemplified by our proteomic deconvolution experiments.

**Supplementary Information:**

The online version contains supplementary material available at 10.1186/s13059-024-03251-5.

## Background

A prominent approach to studying tissue-specific gene expression changes in human development and disease is RNA sequencing (bulk RNA-seq). Tissues, however, usually consist of multiple cell types in different quantities and with different gene expression programs. Consequently, bulk RNA-seq from tissues measures average gene expression across the constituent cells, disregarding cell type-specific changes. The quantification of the cellular composition and cell type-specific expression that underlies bulk RNA-seq data is therefore of pivotal importance to understanding disease mechanisms and identifying potential therapeutic interventions [1].

A recent technological advancement, single-cell RNA-seq, allows for investigating gene expression in single cells for thousands of individual cells of a given tissue sample in a single experiment. However, while it provides unprecedented insights into single-cell biology, it suffers from severe technical limitations, most notably the presence of zero values in gene expression due to methodological noise, termed as “dropouts” [2]. In addition, the technology is still very costly, which essentially prohibits its application in clinical and diagnostic settings. Bulk RNA-seq, on the other hand, can be performed for a fraction of the cost and is widely used in clinical oncology and drug discovery [3, 4].

Computational inference of cell type fraction and cell type-specific gene expression is a source-separation task, termed as “cell deconvolution” within the context of cell biology. The estimation of cell type-specific gene expression is a well established and challenging problem in the field. Prior work includes but is not limited to TAPE [5], bMIND [6], BayesPrism [7], and CibersortX (CSx) [8]. The basic aim is to provide cell type-specific gene expression information at a group or sample level. The resultant information allows deep biological insights into cell type-specific gene expression and pathway changes from bulk data. For cell deconvolution, recent computational methods utilize single-cell sequencing data to create simulated references with known fraction and expression for training [9]. While this approach achieves good deconvolution results, its performance suffers from the substantial domain shift between single-cell RNA-seq training (reference) data and the bulk RNA-seq target data. Domain refers to the statistical distribution of the source of a dataset [10]. Domain shift refers to a change in the statistical distribution of samples, which can be due to covariate shift, the presence of open sets, or both. In gene expression datasets, the covariate shift between real data and simulated datasets occurs due to changes in cell type-specific gene expression and can arise from different dropout rates and tissue conditions, for instance. When domain shifts have purely technical reasons, they are often termed batch effects. CSx [8] has previously approached the problem of batch effect removal between single cell gene expression datasets [11], using Combat [12] to remove changes in cell type-specific gene expression between a single-cell reference signature matrix and bulk RNAseq data. Open sets may occur when new cell types are encountered during test time, such as the presence of differing cell lineages [13]. Since cells go through different differentiation states, domain shift between real data and simulations may be a combination of both, the covariate shift and presence of open sets. Among many possible sources of domain variation, the most prevalent might be the presence of batch effects that refer to technological differences between two sequencing experiments and gene expression differences of biological nature.

In this work, we first formally define the task of cell deconvolution and outline the hypothesis that semi-supervised consistency regularization should improve bulk RNA-seq deconvolution when learning from single cell RNA-seq data. We then provide evidence that two novel deep learning algorithms with semi-supervised consistency regularization outperform competing state-of-the-art algorithms in deconvolution, both on a cellular and gene expression level, across a wide range of datasets. On the datasets with ground truth flow cytometry cell type proportions, DISSECT achieves consistently better Jensen-Shannon distance (*JSD*): 0.063 ± 0.015 and root mean squared error (*rmse*): 0.021 ± 0.019. In addition, DISSECT shows state-of-the-art gene expression deconvolution performance, achieving the best sample- and gene-wise correlations. Our algorithm can easily be adapted to other biomedical data types, as exemplified by our bulk proteomics and spatial expression deconvolution experiments.

## Results

In this section, we first formally define the cell deconvolution task, then present our hypothesis and DISSECT deep learning models, and compare DISSECT’s performance to other state-of-the-art deconvolution algorithms.

### Task of cell deconvolution

Given an $m \times n$ gene expression matrix $\textbf{B}$ consisting of *m* bulk gene expression vectors measuring *n* genes, the goal of deconvolution is to find an $m \times c$ matrix $\textbf{X}$ of cell type fractions, where *c* is the number of cell types present in bulk samples such that,

1 $$\begin{aligned} \textbf{B} = \textbf{X}\textbf{S}, \end{aligned}$$

where fractions and gene expression satisfy non-negativity $0 \le \textbf{X}_{ik},$ and $0 \le \textbf{S}_{kj}$, $\forall i \in [1,m], \forall j \in [1, n]$ and $\forall k \in [1,c]$ and sum-to-1 criterion, i.e., $\sum \limits _{k=1}^{c}\textbf{X}_{ik}$ = 1, $\forall i \in [1,m]$. Here, $\textbf{S}$ is known as the signature matrix and is unobserved. Each row $\textbf{S}_{k\cdot }$ is a gene expression profile (or signature) of cell type *k*. To utilize a reference based framework, $\textbf{S}$ can be replaced with $\textbf{S}_{ref}$ derived from a single-cell experiment by identifying the most representative cell type specific gene expression [8].

The problem of reference-based cell deconvolution can alternatively be formulated as a learning problem, where a function *f* such that $f(\textbf{B}) = \textbf{X}$ is learnt. Since only $\textbf{B}$ is available and $\textbf{X}$ is generally unknown, simulations from a single-cell reference can be used to learn *f*. Clearly, from the above formulation of the cell deconvolution task, it is reasonable to assume linearity of deconvolution, i.e., each bulk mixture is a linear combination of expression vectors of cells spanned with corresponding cell type fractions. Thus, as defined previously in Scaden [9], multiple single cells can be combined in random proportions to generate training examples $\textbf{B}^{\text {sim}}$ and $\textbf{X}^{\text {sim}}$, where each row of $\textbf{B}^{\text {sim}}$ is defined as,

$$\begin{aligned} \textbf{B}^{\text {sim}}_{i \cdot } = \sum \limits _{k=1}^{c} \sum \limits _{l=1}^{\alpha _{k,i}} \textbf{e}^k_{l}, \end{aligned}$$

where $\textbf{e}^k_{l}$ is the expression vector of cell *l* belonging to cell type *k*, and $\alpha _{k,i}$ is the number of cells belonging to cell type *k* sampled to construct $\textbf{B}^{\text {sim}}_{i \cdot }$. Correspondingly, each element of $\textbf{X}^{\text {sim}}$ is the proportion of a cell type *k* in that sample *i* and is defined as,

$$\begin{aligned} \textbf{X}^{\text {sim}}_{ik} = \frac{\alpha _{k,i}}{\sum \limits _{k=1}^{c}\alpha _{k,i}}, \end{aligned}$$

In this case, since each simulated sample has a distinct signature (i.e., gene expression profile), $\textbf{S}$ is a three dimensional matrix with each element $\textbf{S}_{kji}$ denoting gene expression of gene *j* in cell type *k* for sample *i*. It is computed as following,

$$\begin{aligned} \textbf{S}^{\text {sim}}_{k\cdot i} = \frac{\sum \limits _{l=1}^{\alpha _{k,i}} \textbf{e}^k_{l}}{\alpha _{k,i}}. \end{aligned}$$

The predictor *f*, learned from a simulated dataset, can then be applied to $\textbf{B}$ to estimate $\textbf{X}$. Note that, the genes expressed may differ between vectors $\textbf{e}_l$ and $\textbf{B}$ and as such before learning function *f*, each $\mathbf {e^k_l}$ is subsetted to include genes common with $\textbf{B}$. This is the reason why this learning problem is transductive and a separate model needs to be reconstructed for each $\textbf{B}$.

#### Exploiting the linearity of deconvolution

The deconvolution task is to learn a cell type-specific gene-expression matrix (or signature matrix) $\textbf{S}$, which serves to accurately predict cell fractions and their corresponding gene expression from a bulk gene expression matrix $\textbf{B}$. The actual mixing process of cells to form a tissue is assumed to be linear and, as such, the relationship between $\textbf{B}$ and $\textbf{S}$ is linear. However, $\textbf{S}$ is unobserved, and the deconvolution algorithm is learned using simulations. This learning process involving simulations is highly dependent on the reference being the single-cell dataset used to generate simulations, and is subjected to an inherent strong domain shift [14]. To address this, we hypothesize that a consistency-based regularization penalizing the non-linearity of mixtures of real and simulated samples would result in a mapping $\hat{f}$ that is closer to true mapping *f*. Non-linearity of mixtures of real and simulated samples refers to the violation of Eq. 4, defined later, for estimated $\textbf{X}_{i\cdot }, \textbf{X}^{\text {sim}}_{i\cdot }$ and $\textbf{X}^{\text {mix}}_{i\cdot }$ using mapping *f*.

#### Consistency regularization

Consider that $\textbf{B}$ represents gene expression matrices of real (test) bulk RNA-seq that we want to deconvolve and and $\textbf{B}^{\text {sim}}$ represents gene expression matrix of simulated bulk samples. The number of rows (representing samples) in these two matrices may differ. To simplify the notation, we use the same index *i* to denote indices for real bulk samples, simulations ($\text {sim}$) and their mixtures ($\text {mix}$, defined further). Given a true bulk RNA-seq sample $\textbf{B}_{i \cdot }$, and a simulated sample $\textbf{B}^{\text {sim}}_{i\cdot }$ with paired proportions $\textbf{X}^{\text {sim}}_{i\cdot }$ defined over a common set of genes, we can generate a mixture $\textbf{B}^{\text {mix}}_{i\cdot }$ such that

2 $$\begin{aligned} \textbf{B}^{\text {mix}}_{i\cdot } = \beta \textbf{B}_{i\cdot } + (1-\beta )\textbf{B}^{\text {sim}}_{i\cdot }, \end{aligned}$$

Which gives us the relation

3 $$\begin{aligned} \textbf{X}^{\text {mix}}_{i\cdot }\textbf{S}^{\text {mix}}_{\cdot i\cdot } = \beta \textbf{X}_{i\cdot } \textbf{S}_{\cdot i \cdot } + (1-\beta ) \textbf{X}^{\text {sim}}_{i\cdot } \textbf{S}^{\text {sim}}_{\cdot i \cdot }. \end{aligned}$$

where $\textbf{X}_{i\cdot }$ represents cell fractions of sample *i* and where $\beta \in [0,1]$. Cell types are characterized by a few marker genes that are invariant across cell states and even across tissues [15]. A network that accurately predicts cell type fractions based on gene expression of simulated or real bulk RNA-seq data would thus have to learn them. In the estimation of cell type fractions, we therefore assume that the expression of these marker genes should be identical in signatures ${\textbf {S}}^\text {mix}_{\cdot i\cdot }, {\textbf {S}}_{\cdot i\cdot } \text { and } {\textbf {S}}^{{\textbf {sim}}}_{\cdot i\cdot }$. Hence,

4 $$\begin{aligned} \textbf{X}^{\text {mix}}_{i\cdot } = \beta \textbf{X}_{i\cdot } + (1-\beta )\textbf{X}^{\text {sim}}_{i\cdot }, \end{aligned}$$

Equation 4 serves as the formulation to generate pseudo ground-truths for these mixtures during learning, and it enables the use of consistency regularization without having to explicitly estimate signatures. In an iterative learning process $\textbf{X}_{i\cdot }$ can be replaced with predictions of the algorithm from the previous iteration. Naturally, it is also possible to only mix real samples with each other. The number of bulk RNA-seq samples is, however, considerably lower (tens to hundreds) than the amount of single-cells present in a single-cell experiment (thousands or more). Equation 4 allows to generate pseudo ground truth proportions for mixtures $\textbf{B}^{\text {mix}}_{i\cdot }$ at each step of learning cell type fractions, while Eq. 3 allows to generate pseudo ground truth signatures at each step of learning gene expression profiles.

### Network architecture and learning procedure

**Fig. 1:**
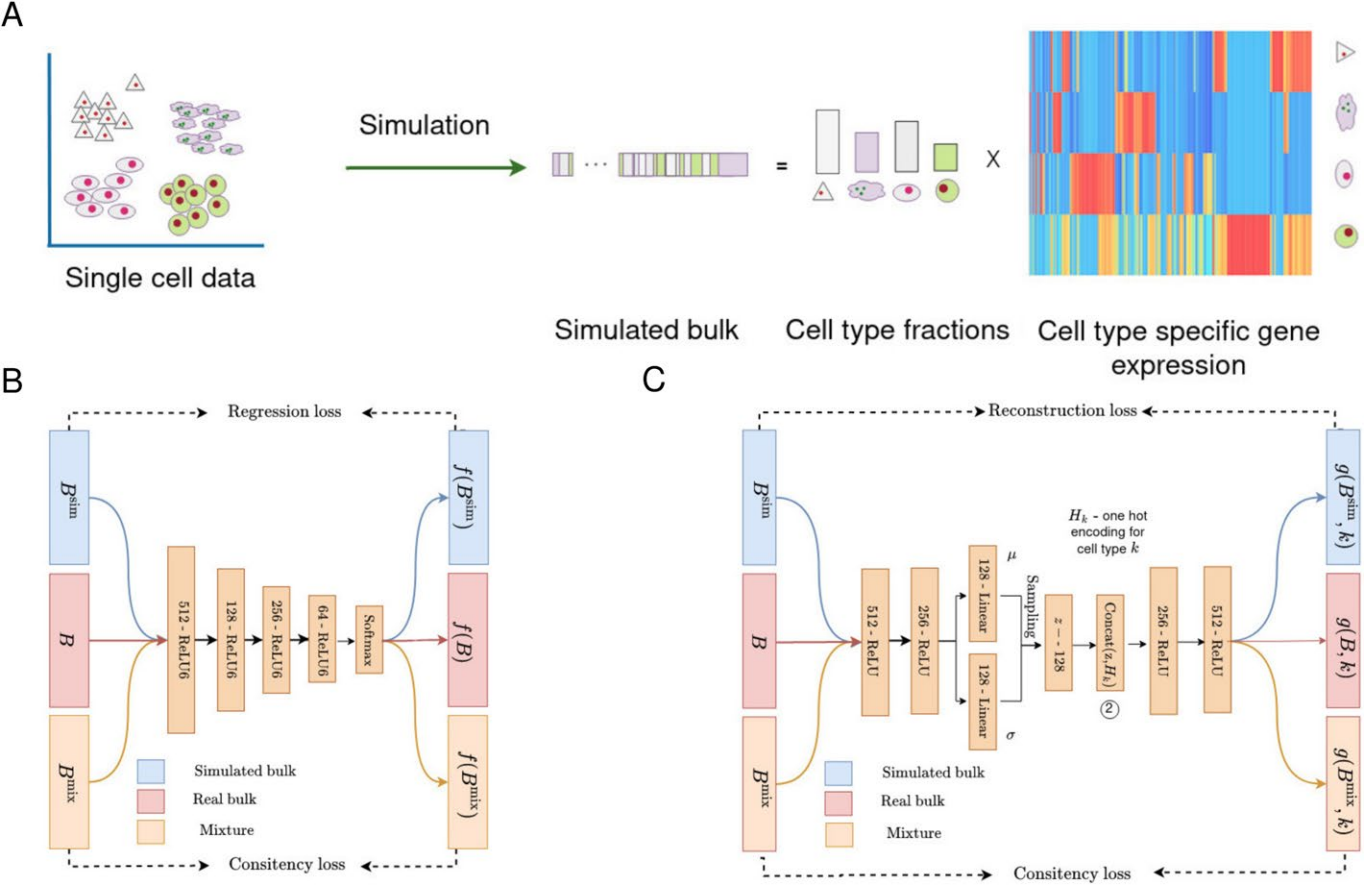
**A** Illustration of the simulation procedure using reference single-cell data. The figure shows the simulation of one sample which consists of cell type fractions, simulated gene expression and cell type specific gene expression profiles (i.e., signature matrix). **B** Detailed overview of an MLP used to estimate cell type fractions. **C** Overview of an autoencoder used to estimate cell type specific gene expression profiles

We approach the two tasks, estimation of cell type fractions and estimation of gene expression profiles per cell type as two different tasks because of their differing assumptions. For the estimation of cell type fractions, we assume that signatures are identical for each sample, both simulated and bulk, while to estimate gene expression, we relax this condition and involve complete consistency regularization (Eq. 3). An illustration of the method is presented in Fig. 1.

#### Estimation of cell type fractions

The underlying algorithm of the first part of our deconvolution method is an average ensemble of multilayered perceptrons (MLPs). The ensembling is performed to reduce the variance by averaging different runs [16]. Each MLP consists of the same architecture initialized with different weights. Each MLP has an architecture: Input (# genes) - ReLU6 (512) - ReLU6 (256) - ReLU6 (128) - ReLU6 (64) - Linear (# cell types) - Softmax. ReLU6 (output of ReLU activation clipped by a maximum value of 6) [17, 18] was chosen out of tested activations over grid search on (Linear, ReLU, ReLU6, Swish [19]). The final application of a softmax activation function allows to achieve the non-negativity and sum to 1 criteria of deconvolution. We train the network with batch size 64 to minimize the loss function per batch defined below with an Adam Optimizer with initial learning rate of $1e-5$.

5 $$\begin{aligned} \mathcal {L}_{\text {total}} \left(\textbf{X}^{\text {sim}}_{i\cdot }, f\left({\textbf{B}}^{\text {sim}}_{i\cdot }\right),\textbf{X}^{\text {mix}}_{i\cdot }, f\left(\textbf{B}^{\text {mix}}_{i\cdot }\right)\right){} & {} = \mathcal {L}_{\text {KLdivergence}}\left(\textbf{X}^{\text {sim}}_{i\cdot }, f\left({\textbf{B}}^{\text {sim}}_{i\cdot }\right)\right) \nonumber \\{} & {} \quad + \lambda _1*\mathcal {L}_{\text {cons}}\left(\textbf{X}^{\text {mix}}_{i\cdot }, f\left(\textbf{B}^{\text {mix}}_{i\cdot }\right)\right), \end{aligned}$$

where $\mathcal {L}_{\text {KLdivergence}}(\cdot , \cdot )$ is the Kullback-Leibler divergence and $\mathcal {L}_{\text {cons}}(\cdot , \cdot )$ is the consistency loss defined as:

$$\begin{aligned} \mathcal {L}_{\text {cons}}\left(\textbf{X}^{\text {mix}}_{i\cdot }, f\left(\textbf{B}^{\text {mix}}_{i\cdot }\right)\right) = \left\|\textbf{X}^{\text {mix}}_{i\cdot } - f\left(\textbf{B}^{\text {mix}}_{i\cdot }\right)\right\|^2_2, \;\text {and} \end{aligned}$$

$$\begin{aligned} \textbf{X}^{\text {mix}}_{i\cdot } = \beta f(\textbf{B}_{i\cdot }) + (1-\beta )\textbf{X}^{\text {sim}}_{i\cdot }. \end{aligned}$$

To generate mixtures, for each batch, we sample $\beta$ uniformly at random for Eq. 4. The interval [0.1, 0.9] was chosen for the uniform distribution to allow for at least some real and some simulated gene expression in the mixture. Since the number of simulations is generally larger (in our experiments, set to 1,000 times the number of cell types) than that of real data, we sample real data to create additional bulk samples, $\textbf{B}_{i\cdot }$, until the size equals that of the simulated data, $\textbf{B}^{\text {sim}}_{i\cdot }$. This pair of data together with simulated proportions, $\textbf{X}^{\text {sim}}_{i\cdot }$, is then used to create training batches of size 64. For every batch, we generate mixtures according to Eq. 2.

Our loss is inspired by MixMatch [20], which uses unlabelled samples to mix up and match sample predictions. Our adaptation in Eq. 5 addresses the limited samples available from true bulk RNA-seq, unavailability of sample fractions and is derived from the definition of the task itself. In essence, Eq. 5 integrates domain knowledge into the objective.

To avoid a scenario where the network does not learn and outputs predictions such that $f(\textbf{B}^{\text {mix}}_{i\cdot }) = f(\textbf{B}^{\text {sim}}_{i\cdot }) = f(\textbf{B}_{i\cdot })$, which is a solution to Eq. 4, we first let the model learn purely from simulated examples. This allows the model to learn meaningful expression profiles to achieve accurate results on simulated examples. We selected $\lambda _1$ based on a grid search over constant and step-wise functions. We adopt a step-wise function for $\lambda _1$, given as:

$$\begin{aligned} \lambda _1 = \left\{ \begin{array}{ll} 0 &{} \text {if step} \le 2000, \\ 15 &{} \text {elif}\ 2000 \le \text {step} \le 4000, \\ 10 &{} \text {else.} \end{array}\right. \end{aligned}$$

We train the network for a predefined number of steps as opposed to epochs, since it is possible to generate infinitely many simulated samples without increasing the intrinsic dimensionality of the data. In our experiments, we limit the number of steps to 5000 as found optimal in Scaden [9].

##### Estimation of per sample cell type specific gene expression profiles

Estimation of cell type fractions from bulk RNA-seq requires an assumption that signatures of cell types are shared across single cell and bulk RNA-seq. However, cell type gene expression profiles (at least for genes that are not invariant across tissue states) may differ between samples. Previously, works such as CSx [8] and TAPE [5] have explored utilizing cell type fractions to estimate gene expression per sample. Here, we make use of a $\beta$-variational autoencoder with standard normal distribution as prior to estimate average gene expression of the different cell types from bulk RNA-seq expression levels. To jointly train the network on all cell types, we condition the decoder (at its input layer) with cell type labels. This allows for training a single model to estimate gene expression of each cell type for a sample. To make use of bulk RNA seq during the training, we regularize the reconstruction loss with a consistency loss defined over per cell type signature. Denoting *f* as before and $g(\cdot , k)$ as the output of the autoencoder with condition *k* (corresponding to cell type label) on the decoder input, this consistency loss is defined as:

$$\begin{aligned} \mathcal {L}_{\text {cons}}^{\text {VAE}}\left(f, g, \textbf{B}_{i \cdot }^{\text {mix}}, \textbf{B}_{i \cdot }, \textbf{X}^{\text {sim}}_{i\cdot }, {\textbf {S}}^{\text {sim}}_{ki\cdot }\right)&= \left\|f\left(\textbf{B}_{i \cdot }^{\text {mix}}\right)_{k}g \left(\textbf{B}^{\text {mix}}_{i\cdot }, k\right) - \beta f\left(\textbf{B}_{i \cdot }\right)_{k} g(\textbf{B}_{i\cdot }, k)\right. \nonumber \\&\quad - \left.(1-\beta ) \textbf{X}^{\text {sim}}_{i\cdot } {\textbf {S}}^{\text {sim}}_{ki\cdot }\right\|^2_2, \end{aligned}$$

where $\textbf{B}^\text {mix}_i$ is given by Eq. 2, and $f(\textbf{B}_{i \cdot }^{\text {mix}})_{k}$ is the proportion of cell type *k* in sample *i* as estimated during cell type fraction estimation and is fixed during training. In implementation, we replace $f(\textbf{B}_{i \cdot }^{\text {mix}})_{k}$ with $\beta f(\textbf{B}_{i \cdot })_{k} + (1-\beta ) \textbf{X}^{\text {sim}}_{i\cdot }$. Thus, this loss forces the learned signature for cell type *k*, $g(\textbf{B}^{\text {mix}}_{i\cdot }, k)$, to be closer to signatures for both real and simulated bulk samples. This loss function makes the assumption that mixing two bulk samples is similar to mixing individual cell type specific signatures that constitute those bulks. We added this loss function with a regularization parameter $\lambda _2$ (with default value 0.1) to the loss of the standard $\beta$-variational autoencoder (the weight on the KL divergence, denoted as $\beta ^{\text {VAE}}$, is set to 0.1 by default). The total loss function sums up to:

$$\begin{aligned} \mathcal {L}^{\text {VAE}}_{\text {total}}\left(f, g, \textbf{B}^{\text {sim}}_{i\cdot }, \textbf{B}_{i \cdot }^{\text {mix}}, \textbf{B}_{i \cdot }, \textbf{X}^{\text {sim}}_{i\cdot }, {\textbf {S}}^{\text {sim}}_{ki\cdot }\right)&= \left\|\textbf{S}^{\text {sim}}_{ki\cdot } - g\left(\textbf{B}^{\text {sim}}_{i\cdot }, k\right)\right\|^2_2 \\&\quad + \lambda _2 \mathcal {L}_{\text {cons}}^{\text {VAE}}\left(f, g, \textbf{B}_{i \cdot }^{\text {mix}}, \textbf{B}_{i \cdot }, \textbf{X}^{\text {sim}}_{i\cdot }, {\textbf {S}}^{\text {sim}}_{ki\cdot }\right) \\&\quad + \beta ^{VAE} \mathcal {L}_{\text {KLdivergence}} (\mathcal {N}(\mu , \sigma ), \mathcal {N}(0,1)), \end{aligned}$$

where $\mathcal {N}(0,1)$ is standard normal distribution, and $\mu \text { and } \sigma$ are the empirical mean and standard deviation estimated from the output of the encoder. Both the encoder and decoder consist of two hidden layers. Under default settings used throughout this work, we train the network to minimize the loss function with an Adam optimizer with initial learning rate of $1e-3$, and the values for hyperparameters $\lambda _2$ and $\beta ^{\text {VAE}}$ are respectively 0.1 and $1e-2$. The network is trained for 5000 $\times k$, *k* being the number of cell types.

### Estimation of cell type fractions and comparison with flow cytometry

To quantitatively assess the deconvolution algorithm, we first deconvolve six different peripheral blood mononuclear cells (PBMC) bulk datasets for which cell type proportions have already been quantified using flow cytometry (Additional file 1: Table S1). To evaluate deconvolution performance, we utilize root-mean-squared error (*rmse*) and Pearson correlation (*r*) for cell type-wise comparisons and Jensen-Shannon distance (*JSD*) for sample-wise comparisons between estimated fractions and ground truth proportions. The evaluation metrics are defined in the “Evaluation metrics” section. To evaluate our approach, we compared it to state-of-the-art deconvolution methods, MuSiC [21], CSx [8], Scaden [9] and TAPE (TAPE-O and TAPE-A) [5], BayesPrism and BayesPrism-M [7], and bMIND [6]. MuSiC and CSx were chosen for their best performances in benchmarking studies [22, 23]. Scaden and TAPE are selected as both are deep learning-based deconvolution approaches, the latter of which, TAPE-A, performs an adaptation of the network weights for test samples. Since deconvolution is linear, we also considered linear MLPs as a deconvolution algorithm. Further details can be found under the “State of the art” section.

We utilize the *PBMC8k* single cell RNA-seq dataset as reference (Additional file 1: Table S2) for all methods. The first two principal components of combined simulated and real PBMC datasets are visualized in Additional file 2: Fig. S1A, illustrating a domain shift between datasets.

**Fig. 2:**
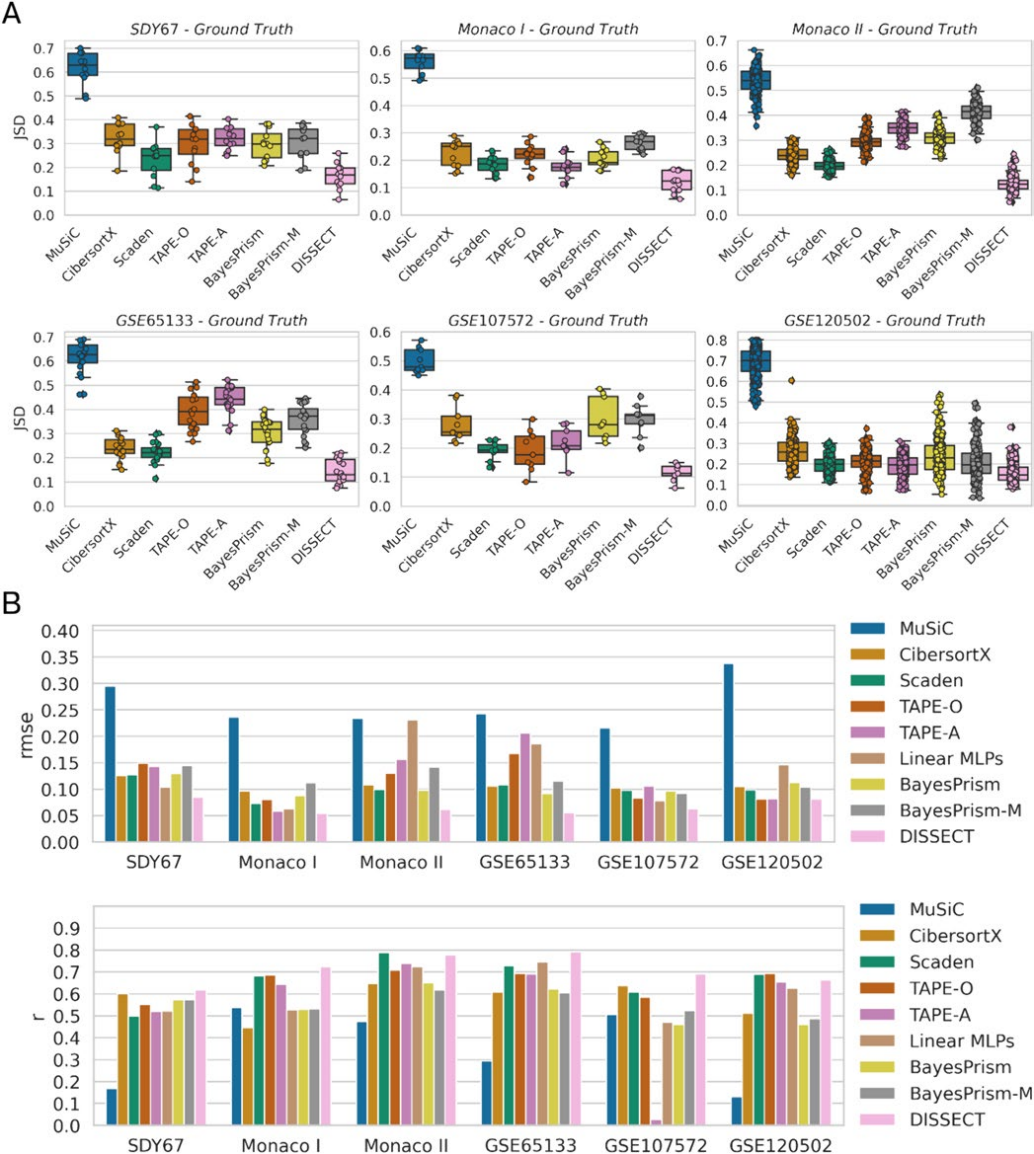
Evaluation of deconvolution algorithm on six datasets with ground truth information. **A** Per-sample Jensen-Shannon divergence (*JSD*). Each plot corresponds to a dataset. From left to right and top to bottom: *SDY67*, *Monaco I*, *Monaco II*, *GSE65133*, *GSE107572*, and *GSE120502*. **B** Root mean-squared-error (*rmse*, top) averaged over cell types for each of the dataset. Datasets are listed on *x*-axis. Pearson’s correlation (*r*, bottom) averaged over cell types

For each dataset, DISSECT always obtained the best *JSD* across all datasets (Fig. 2A), leading to an average improvement over the second-placed algorithms of 6 percentage points. On the *GSE65133* dataset, for instance, DISSECT outperforms second-paced Scaden by 8 percentage points (DISSECT: *JSD* = 0.145, Scaden: *JSD* = 0.222). Similarly, DISSECT always obtains the best *rmse* across all datasets and improves over second-placed algorithms by 2 percentage points, on average (Fig. 2B). In addition, it achieved the best *r* on 4 out of 6 datasets (Fig. 2B).

Furthermore, we computed *macro-* level *r* and *rmse* by computing the metrics without making a distinction of cell types as performed previously in [9]. Note that in this setting, *JSD* remains unaffected as it is a sample-level metric and is therefore excluded. We observe that DISSECT achieves consistently best *rmse* across all datasets while achieving best *r* on 5 out of the 6 datasets (Additional file 2: Fig. S1).

Since MuSiC can take advantage of multi-sample references, we also evaluated MuSiC using blood data from the Immune Cell Atlas (ICA) (Additional file 1: Table S2). We also evaluated MuSiC with pre-selected marker genes (MuSiC-M) that were selected by CSx. MuSiC-M showed increased performance in 4 out of 6 datasets (Additional file 2: Fig. S2A-B). MuSiC also shows improved performance in the multi-sample setting in both rmse (Additional file 2: Fig. S2A) and r (Additional file 2: Fig. S2B). DISSECT still reaches best performance in *rmse* (on average 8 percentage points better) and *r* (on average 13 percentage points better) across all datasets.

Next, we evaluated the cell fraction deconvolution performance on the *Monaco I* (Additional file 1: Table S1) dataset, which contains several closely related and rare cell types and constitutes a relatively hard cell deconvolution task, using *Ota* dataset (Additional file 1: Table S1). With a correlation of 0.6, DISSECT’s average performance is 14 percentage points better than the second placed Scaden (Additional file 1: Table S3), while Scaden’s average RMSE was marginally (1 percentage point) better than second placed DISSECT (Additional file 1: Table S4). To validate that the performance improvement in DISSECT is due to the semi-supervised learning and consistency loss, we performed an ablation study on data *SDY67* by successively and cumulatively removing components of the algorithm and testing it again. The following components were removed successively: consistency regularization, KL Divergence loss (mean squared error instead), and the nonlinear activation function (identity function instead). The ablation results are shown in Additional file 1: Table S5.

In summary, these results provide strong evidence that DISSECT consistently outperforms current state-of-the-art cell type deconvolution algorithms across six different datasets with ground truth information.

### Consistency of predictions and relationship between cell type fractions and biological phenotypes

To further corroborate the above results, we evaluate DISSECT’s performance on three datasets that do not have paired flow cytometry data. In this section, we compare to other established biological facts as well as divergences over different reference single-cell datasets. The bulk datasets together with literature-based expected biological relationships of cell types are listed in Additional file 1: Table S1.

#### Brain

The *ROSMAP* dataset consists of 508 bulk RNA-seq samples from the dorsolateral prefrontal cortex (DLPFC) of patients with Alzheimer’s disease (AD) as well as non-AD samples (Additional file 1: Table S1). For 463 of these samples, Braak stages of disease severity have been quantified. Correspondingly, single-nuclei RNA-seq (snRNA-seq) for 48 individuals from the same cohort is available [24]. For 41 of these samples, cell type fractions based on immunohistochemistry (IHC) from a previous work exist [25]. It should be noted that IHC was performed for all neurons and as a result, comparison with respect to excitatory vs inhibitory neurons was not possible. Here, we consider two biological ground truths: first is the ratio of excitatory neurons to inhibitory neurons (Additional file 1: Table S1), and second is the neurodegeneration, or the loss of neurons with increasing Braak Stages [26]. We deconvolved *ROSMAP* using the *Allen Brain Atlas* reference (Additional file 1: Table S2).

**Fig. 3:**
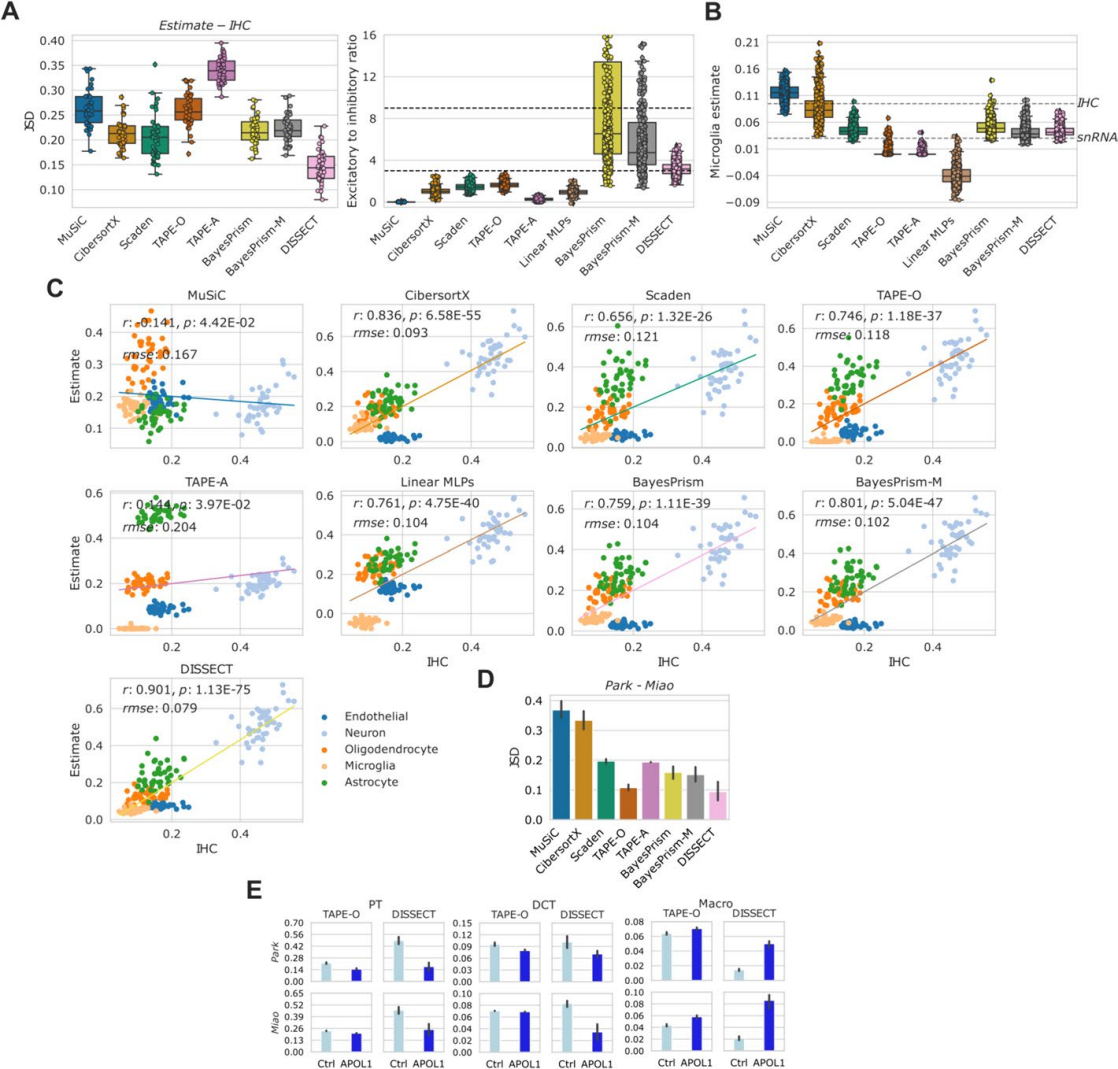
**A** Left: Box-plots showing *JSD* between estimated fractions and IHC based cell type proportions from 41 individuals from *ROSMAP*. Right: Ratio of excitatory to inhibitory neurons computed from *ROSMAP*. Expected ratios lie between 3:1 and 9:1 as indicated by dashed lines. **B** Boxplots showing microglia proportion as estimated by different methods. Median proportions of microglia estimated using snRNA-seq and IHC are labeled. **C** Correlations between estimates (*y-axis*) and IHC cell type proportions (*x-axis*). **D** *JSD* between predicted proportions from *Kidney* between experiments with *Miao* and *Park* as references. **E** Predictions from TAPE-O and DISSECT from *Kidney*. From left to right: Proximal tubule (PT), ductal convoluted tubules (DCT), and macrophages (Macro). Each row indicates a reference. Error bars show standard deviations, while height of the bars shown mean prediction

We computed the *JSD* between the estimated fractions and IHC cell type proportions. DISSECT estimated fractions had the best average *JSD*s and provides the expected excitatory-inhibitory neuron ratio of (3:1–9:1), while other methods generally underestimated this ratio (Fig. 3A). All methods recover a negative correlation between increasing Braak stages and the fraction of neurons (Additional file 2: Fig. S3).

Previously, it has been noted that snRNA-seq and IHC data provide different estimates for some cell types, notably microglia and endothelial cells [25]. It is interesting to observe that DISSECT and Scaden were the only methods where the estimates of microglia resembled closely those obtained from snRNA-seq and IHC data (Fig. 3B). We also computed *r* and *rmse* between the IHC cell type proportions and estimated fractions (Fig. 3C). With a correlation *r* of 0.901 DISSECT proved to be 14 percentage points better than the second-placed linear MLP. DISSECT also displayed the best *rmse* at 0.079.

Overall, the comparison to IHC and snRNA-seq ground truth information for the ROSMAP data further strengthens our claim that consistency regularization with DISSECT robustly improves cell deconvolution.

#### Pancreas

The *GSE50244* bulk RNAseq dataset consists of 89 pancreas samples from healthy and type 2 diabetes (T2D) individuals (Additional file 1: Table S1). For 77 of these samples, hemoglobic 1C levels are available as ground truth information. We performed the deconvolution using three single-cell reference datasets *Baron*, *Segerstolpe*, and *Xin* (Additional file 1: Table S2). Both *Baron* and *Segerstolpe* datasets contain alpha, beta, gamma, delta, acinar, and ductal cell types. While only alpha, beta, gamma, and delta cell types were present in the *Segerstolpe* dataset. To measure the consistency of deconvolution algorithms, we measured *JSD*s between estimated fractions using each of the three references (Additional file 2: Fig. S4A). While several methods showed considerable divergences, indicating reference-dependent deconvolution results, DISSECT displayed the most consistent results with a *JSD* of $\sim$0.1–0.2 across the three pairs. In terms of recovery of significant negative correlations between the estimated fractions of beta cells and hemoglobin 1C (hba1c) levels, DISSECT provided highly significant correlations of between − 0.45 and − 0.47 across the three references (Additional file 2: Fig. S4B). These results further suggest that DISSECT is both precise and robust in cell type deconvolution on real data and is comparatively less affected by the choice of single-cell reference.

#### Kidney

The *GSE81492* dataset consists of 10 kidney samples of APOL1 mutant mice, which is a mouse model of chronic kidney disease (CKD) (Additional file 1: Table S1). We deconvolved the dataset using two single cell references: *Miao* and *Park* (Additional file 1: Table S2). Similar to our experiments on the pancreas tissue, we computed *JSD* between the estimated cell type fractions from the two references. DISSECT provided the best average *JSD* (0.09) out of all considered methods (Fig. 3D). We further compare the methods on the recovery of expected relation of cell type fractions with the biological phenotype (Additional file 1: Table S1). Figure 3E compares two best methods on *JSD*, DISSECT, and TAPE-O, while Additional file 2: Fig. S5 presents these results on all cell types for all methods. It is known that CKD results in the decrease in proximal tubule cells (PT) and distal convoluted tubules (DCT). Cell type fractions estimated with DISSECT showed a significant loss of PTs and DCTs and a corresponding increase in macrophages, while TAPE-O provided much smaller differences between the control and CKD model (Fig. 3E). PTs are the most abundant cell type in kidney making up around 50% of a mouse kidney [27]. DISSECT correctly estimates the high abundance of PTs in healthy kidney, while TAPE-O underestimates them (Fig. 3E).

In summary, it is noteworthy that DISSECT shows state-of-the-art precision and robustness in cell type deconvolution across various ground truth information and 9 datasets, including PBMC, brain, pancreas, and kidney bulk RNA-seq samples. DISSECT also shows superior robustness to the choice of single cell reference.

### Application to proteomics and spatial transcriptomics

It is conceivable that DISSECT’s consistency regularization for bulk RNA-seq cell type deconvolution should also lend itself to other biomedical datatypes in which domain shifts might be a problem. Applications might include, for example, the deconvolution of spatial transcritomic (ST) and bulk proteomic data with supra-cellular resolution. In order to evaluate these potential use-cases, we performed deconvolution of spatial transcriptomics and proteomics samples. Here, our aim is to test the hypothesis of applicability of DISSECT on these data modalities and we do not intend to perform an exhaustive comparison to multiple methods developed for these modalities. For comparisons on spatial transcriptomics, we consider four state-of-the-art spatial deconvolution methods, RCTD [28], Cell2location (C2L) [29] as shown to perform among the best in the benchmarking study [30]. We also include SONAR [31] and CARD [32], both of which can utilize spatial information. For comparisons on proteomic deconvolution, we consider the tested bulk deconvolution methods.

#### Spatial transcriptomics

We evaluated DISSECT on the task of spatial deconvolution using mouse brain and human lymph node samples (Additional file 1: Table S1). As a ground truth, we considered relationships with biological phenotypes in line with our application of kidney and pancreas datasets (Additional file 1: Table S1). Due to the spatial nature of the ST, we could verify the recovery of neuronal layers in brain (Additional file 2: Fig. S6) and discernment of germinal centers in lymph node (Additional file 2: Fig. S7). DISSECT performs on par with C2L and RCTD on both datasets. The results are provided and discussed in detail in the Additional file 2: Supplementary Note.

#### Proteomics

To compare the ability of the tested deconvolution methods to recover cell type proportions from proteomics mixtures, we utilized 50 human brain samples (Additional file 1: Table S1). We applied each deconvolution method on these samples using the Allen Brain Atlas reference (Additional file 1: Table S2). Compared to other methods, DISSECT recovered excitatory neurons to be the expected majority population in both datasets while maintaining the excitatory to inhibitory neuron ratio to be around expected range of (3:1–9:1) (Additional file 2: Fig. S8). These results strongly suggest that DISSECT reaches state-of-the-art performance on proteomic cell type deconvolution and might be applicable to other biomedical data types.

### Evaluation of DISSECT under domain shifts

To assess the impact of consistency regularization on the performance of DISSECT and other algorithms, we used *Ota* dataset (Additional file 1: Table S1). Using this dataset in a dynamic domain shift setup (see the “Domain shift experimental setup” section), we evaluated the performance of deconvolution methods. We also included DISSECT without consistency (DISSECT w/o consistency) to asses the impact of semi-supervised learning under varying shifts. The performance of all methods dropped significantly for test sets with domain shifts (Additional file 2: Fig. S9). However, the drop in performance was much lower for DISSECT than other methods. Furthermore, a clear advantage of semi-supervised learning with consistency regularization is observed in comparison to DISSECT without consistency, especially in terms of *rmse*.

### Estimation of cell type-specific gene expression

So far, we have shown that DISSECT can reliably deconvolve cell fractions. In this section, we focus on the deconvolution and inference of cell type-specific gene expression from bulk RNA-seq mixtures using our novel conditional autoencoder based algorithm (Fig. 1). While we were able to use ground truth flow cytometry data for the evaluation of cell fractions, no such gold-standard is available for cell type-specific gene expression information. In consequence, we measure DISSECT’s gene expression inference performance on simulated bulk RNA-seq data. To maintain a domain shift between the training and test datasets, we simulated data for training and testing using different single-cell datasets. We compared the performance of DISSECT with that of TAPE-A, bMIND, and BayesPrism, all of which can infer cell type-specific gene expression per sample. We simulated bulk samples from one of the four reference single-cell PBMC datasets listed in Additional file 1: Table S2 and created training simulations from the remaining three. Simulations from each single-cell dataset consisted of 6000 samples. To evaluate the performance of DISSECT and other methods, we compared the true and estimated gene expression profiles of each cell type for each simulated sample (sample-wise) and for each gene (gene-wise) using Spearman correlation. These sets of results were aggregated across cell types and averaged. DISSECT displays the best sample- and gene-wise correlations in 6 out of 8 experiments, outperforming TAPE-A by 0.025 ± 0.023 in the sample-wise comparisons and by 0.012 $\pm$ 0.029 in the gene-wise comparisons (Table 1). Moreover, DISSECT exhibited an improvement in both sample and gene-wise metrics, exemplifying its advantage.

**Table 1:** Spearman correlation between ground truth and estimated gene expression profiles on simulated datasets averaged over samples. The column *Dataset* indicates the single-cell dataset used to create simulations for the test set

Dataset	TAPE-A	bMIND	BayesPrism	DISSECT
*sample-wise* *r*				
PBMC6k	**0.83** $\varvec{\pm }$**0.09**	0.80 ± 0.07	**0.83 ± 0.11**	0.82 ± 0.08
PBMC8k	0.79 ± 0.09	0.80 ± 0.08	0.81 ± 0.09	**0.84** $\varvec{\pm }$**0.11**
DonorA	0.85 ± 0.11	0.84 ± 0.09	0.80 ± 0.09	**0.89** $\varvec{\pm }$**0.10**
DonorC	0.81 ± 0.12	**0.83** $\varvec{\pm }$**0.11**	0.80 ± 0.08	**0.83** $\varvec{\pm }$**0.08**
*gene-wise* *r*				
PBMC6k	0.42 ± 0.14	**0.46** $\varvec{\pm }$**0.14**	0.41 ± 0.14	**0.46** $\varvec{\pm }$**0.15**
PBMC8k	**0.51** $\varvec{\pm }$**0.12**	0.44 ± 0.18	0.45 ± 0.12	0.48 ± 0.14
DonorA	**0.48 ± 0.20**	0.45 ± 0.16	0.46 ± 0.18	**0.48** $\varvec{\pm }$**0.18**
DonorC	0.45 ± 0.11	0.43 ± 0.15	0.45 ± 0.12	**0.49** $\varvec{\pm }$**0.12**

For each dataset, values with the highest mean correlation are displayed in bold font

These results indicate that DISSECT’s consistency regularization robustly performs state-of-the-art cell type-specific gene expression deconvolution.

## Discussion

In this work, we first formally define the task of cell deconvolution and outline the hypothesis that semi-supervised consistency regularization should improve bulk RNA-seq deconvolution when learning from single cell RNA-seq data. We then provide evidence that our novel deep learning-based algorithm, DISSECT, outperforms competing state-of-the-art algorithms in deconvolution, both on a cellular and gene expression level, across many different datasets. This included 6 PBMC datasets with ground truth flow cytometry information and 3 datasets (brain, pancreas, and kidney) with other established biological facts as ground truth information. Across the board, DISSECT provided the best cell type deconvolution results when compared to four state-of-the-art methods, while also being comparatively robust to the choice of single-cell reference. We follow a two-step procedure because the assumptions for each of the algorithms differ, and we do not foresee any significant benefit from iteratively deconvolving cell type fractions and gene expression. In a case study, we also show how our algorithm can easily be adapted to deconvolve cell types of proteomic and spatial expression data. For the spatial transcriptomics data, DISSECT estimates cell type fractions per spot, which are constrained to sum to 1. To be able to estimate the number of cells per cell type for each spot, and to map single cells, DISSECT estimates can be used as a prior for algorithms such as CytoSpace [33]. CytoSpace infers both the number of cells in each spot and solves an optimization problem to map single cells to their spatial locations. To estimate only the number of cells per cell type for each spot, the total number of cells as estimated by CytoSpace can be multiplied with the output of DISSECT. While these results are not exhaustive, they nevertheless show the applicability of DISSECT on other biomedical data types, a research avenue we might pursue in more depth in the future. In addition to DISSECT’s state-of-the-art cell type fraction deconvolution (an average improvement of 0.063 in *JSD* and 0.021 in *rmse* over the state of the art on the datasets with ground truth cell type fractions), it achieved best cell type-specific gene expression deconvolution results in 6 out of 8 comparisons across four simulated datasets with an average improvement of 0.025 in the sample-wise and 0.012 in the gene-wise comparisons.

While we focused on MLPs for the estimation of cell type fractions and an autoencoder for gene expression estimation in this work, consistency regularization might also improve other deconvolution algorithms.

No gold standard ground truth exists for quantitative assessment of estimated cell type-specific gene expression between two conditions for real bulk RNA-seq datasets. This is a limitation of the experimental setup presented for cell type-specific gene expression estimation. A potential solution will be to develop biologically valid benchmark datasets that can be evaluated at scale.

While DISSECT outperforms competing algorithms in cell type fraction and cell type-specific gene expression deconvolution, some results leave room for further improvement. DISSECT accurately distinguishes cell types where the transcriptional difference reflects cell subtypes, for instance PBMCs (CD4 T cells and CD8 T cells), pancreas (pancreatic islets), kidney (tubular epithelial cells), and brain (OPC and oligodendrocytes). However, when estimating granular cell type proportions in the Monaco I dataset, error rates exceeded the ground truth proportions (*rmse*>0.01 for cell subsets present at less than 1%). Therefore, for cell types that make up less than 1% of all cells and cells with very similar gene expression, for instance CD4 T and activated CD4 T cells, deconvolution algorithms should be used with caution. Future research into semi-supervised and contrastive algorithms as well as data augmentation and integration techniques should further enhance DISSECT’s performance on hard deconvolution tasks.

## Conclusions

In conclusion, DISSECT provides a semi-supervised deep learning framework to estimate cell type proportions and per-sample cell type-specific gene expression, is robust across datasets and tissues, and is easily applicable to other data modalities. DISSECT delivers state-of-the-art deconvolution performance, as long as cell types are not too closely related and make up more than 1% of all cells.

## Methods

### Evaluation metrics

To quantitively evaluate estimated cell type fractions across samples, we used two metrics, namely Pearson’s correlation (*r*) and root-mean-squared error (*rmse*). Given *x* and *y* as estimated fractions and ground truth respectively,

6 $$\begin{aligned} r = \frac{cov(x,y)}{\sigma _x, \sigma _y} \end{aligned}$$

7 $$\begin{aligned} rmse = \sqrt{Avg(x}-y)^2 \end{aligned}$$

To compute sample-wise divergences two list of fractions $x_i$ and $y_i$ for the same sample *i*, we used Jensen-Shannon distance (JSD) which is the square root of Jensen-Shannon divergence. *JSD* is given as

8 $$\begin{aligned} JSD(x\Vert y) = \sqrt{\frac{D(x_i\Vert m_i) + D(y_i\Vert m_i)}{2}}, \end{aligned}$$

where $m_i = \frac{(x_i+y_i)}{2}$ and *D* is the Kullback-Leibler divergence.

### State of the art

Here, we briefly detail the state-of-the-art deconvolution approaches. Out of these methods, CSx, TAPE, BayesPrism, and bMIND can also estimate per sample cell type-specific gene expression signatures.

#### MuSiC

MuSiC [21] uses weighted non-negative least squares. MuSiC maintains cross-cell and cross-sample consistencies by appropriately weighting genes based on their informativity during an iterative procedure. We used MuSiC R package (version 1.0.0). Deconvolution using MuSiC was performed according to the authors recommendations. Since MuSiC is a method that utilizes multi-subject scRNA-seq datasets, when available, we used cells from multiple subjects in deconvolution with MuSiC. We used the default hyperparameters to execute MuSiC. For single-cell datasets with multiple donors (Additional file 1: Table S2), we ran MuSiC with single-cell data from all available donors.

#### CSx

CSx [8] is a deconvolution method that addresses domain gap problems with scRNA-seq and bulk samples by aiming to correct batch effects. It uses scRNA-seq to generate a cell type specific signature matrix and uses $\nu$-support vector regression as the underlying algorithm. To construct the signature matrix, we used the following hyperparameters for CSx as recommended by the authors: kappa = 999, *q*-value = 0.01 and number of genes within a range of 300 and 500. The quantile normalization was also disabled. CSx comprises two modes, S- and B-modes, to address the domain gap. S-mode is used when deconvolving with a signature matrix constructed using a scRNA-seq dataset, while B-mode is used when deconvolving with a signature matrix constructed using purified samples. We followed the documentation provided by the authors to run CSx and used the S-mode. CSx can also predict gene expression signatures for each sample for which it uses a non-negative matrix factorization based iterative algorithm. However, CSx only estimates genes likely to be differentially expressed in one of the bulk samples and as such the evaluations for simulations from healthy PBMC single-cells are not possible. We ran CSx through docker container obtained from [34].

#### Scaden

Scaden [9] is an average ensemble of three deep neural networks with different architectures that was developed for cell fraction deconvolution. Each network is trained only on simulated pseudo bulk data generated from an scRNA-seq reference similar to described above. Scaden is provided as a Python package. We used the official Scaden package (version 1.1.2) with the instructions provided by the authors to train the networks.

#### TAPE

TAPE [5] is a fully connected autoencoder where the bottleneck consists of cell type fractions. The architecture of the encoder is similar to the archictecture of Scaden but with CeLU activations. The decoder consists of linear activations and outputs gene expression of the input vector. The adaptive mode of TAPE (TAPE-A) aims at optimizing the network for bulk samples, while the overall mode trains for fractions with an added loss function that reconstructs input bulk expression from fractions. Since TAPE-A reconstructs gene expression from fractions (bottleneck), the signature matrix is visible in the (linear) decoder. To estimate gene expression signatures for each bulk sample, decoder weights are optimized per-sample using an iterative optimization strategy. Network weights are changed during the two modes, we compare with both and refer to TAPE in overall mode as TAPE-O and in adaptive mode as TAPE-A. We used the official scTAPE package (version 1.1.2) implemented in Python.

#### Linear MLPs

The solution to the deconvolution problem could be, in principle, a linear function. For this reason, we also compared to an MLP ensemble that has similar architecture to DISSECT, but in which we replaced all non-linear activations with an identity function and removed the consistency loss.

#### BayesPrism and BayesPrism-M

Primarily a method developed for oncology bulk datasets, BayesPrism [7] is a Bayesian framework to infer cell type fraction and cell type specific per-sample gene expression. It models gene expression as multinonmial distribution and calculates the cumulative posterior across cell states to derive the statistics for individual cell types. To evaluate BayesPrism with preselected marker genes using *select.marker* function. We utilize official implementation of BayesPrism in R (version 2.1.2).

#### bMIND

bMIND [6] is a Bayesian method to infer cell type specific gene expression per sample based on single-cell gene expression for given cell types. Using the prior from single-cell gene expression, bMIND models bulk gene expression as the product of gene expression and cell type fractions as a Bayesian mixed-effects model. bMIND uses cell type fractions as estimated by other deconvolution methods as its input. We used default settings of bMIND in our experiemnts with its R implementation (version 0.3.3).

### Pre-processing and simulations

#### Quality control

Before simulating from reference datasets, we remove cells with less than 200 expressed genes and genes which are expressed in less than 3 cells. Furthermore, we also remove cells expressing more than 4% mitochondrial genes. Thereafter, before each deconvolution, we subset reference and bulk datasets to include only the common genes between the two. This quality control step was identical for all methods.

#### Simulations for deconvolution of bulk RNA-seq samples and proteomics

For deep learning methods, we sampled $\alpha _{k,i}$ uniformly to generate simulations *s.t.* $\sum \limits _{k=1}^c\alpha _{k,i} = 100, \forall i$ if the dataset is single-cell. For experiments on granular level cell types where simulations are done from purified cell samples, we modified the simulation procedure to reflect this. In this case, a simulated sample is given by $\textbf{B}^{\text {sim}}_{i\cdot } = \sum \limits _{k=1}^c \textbf{X}^{\text {sim}}_{ik} \mathbf {b^k_l}$, where $\mathbf {b^k_l}$ is the expression vector of purified sample *l* belonging to cell type *k*. For all experiments, we simulated total $1000 \times c$ simulations where *c* is number of cell types in the reference dataset.

#### Simulations for deconvolution of 10x Visium ST samples

We adjusted simulation procedure to mimic ST datasets. 10x Visium (one of the technologies to generate ST samples) consists of around 10 cells per spot. To reflect this, we simulated between 5 and 12 cells to generate one spot (i.e., $\sum \limits _{k=1}^c \alpha _{ki} \sim [5,12]$). Since ST is much sparser, to generate one spot, we kept between 2 and 6 cell types. Due to sparsity of spots, not all cell types are present in a given spot. To account for this and to make comparison across spots possible, we utilized the outputs of the last layer (before performing softmax operation) and set negative predictions to zero. Thereafter, we re-normalized these absolute scores by such that each prediction sum to one. For all experiments, we simulated total $1000 \times c$ simulations where *c* is number of cell types in the reference dataset.

#### Deconvolution of proteomics data

For deconvolution of proteomics data, it is not valid to mix protein intensities and gene expression due to different normalizations. Instead of mixing simulated samples with real samples, proteomics samples were mixed with each other, i.e., at each training step, $\textbf{B}^{\text {mix}}_{i\cdot } = \beta \textbf{B}_{{r_1}\cdot } + (1-\beta )\textbf{B}_{{r_2}\cdot },$ where $r_1$ and $r_2$ are two randomly selected proteomics samples at the training step.

#### Pre-processing for estimation of cell type fractions

For Scaden, TAPE, linear MLPs, and DISSECT, before passing simulated and real bulk samples to the network, we normalize samples to sum to a million counts (counts per million (CPM)) and log scale them with base 2 after adding 1. CPM normalization was performed to maintain total mRNA expressed per gene to be out of a fixed total gene expression, and CPM is widely used in computational genomics. During training, for each batch, we normalize each sample by *MinMax* scaling. These are standard preprocessing steps [9].

For MuSiC and CSx (under S-mode), data was supplied on a linear scale as suggested in their respective publications and no change was made to the default normalization methods of both [8, 21].

To estimate cell type specific gene expression profiles, we need to maintain relationship between gene expression of individual cell types and simulated bulks, which would be lost if we perform CPM normalization of both simulated samples and corresponding cell type specific gene expression profiles. Hence, instead of performing CPM normalization of simulated bulks, we normalize each test bulk sample to sum to the mean of sums of simulated samples. Furthermore, for estimating cell type specific gene expression, we want to maintain gene level information across samples. To achieve this, instead of normalizing each sample using *MinMax* scaling, we perform *MinMax* scaling globally over all samples.

For TAPE, since the signature matrix is observed in decoder (see the “State of the art” section), preprocessing step is similar to the preprocessing done in estimating cell type fractions.

### Hyperparameters and fine-tuning

We fine tuned the network for activation functions, learning rate, and batch size using randomized search with hyperopt [35] with the root mean squared error as the objective function. The following grids were used for the optimization: activations = [linear, ReLU, ReLU6, Swish], learning rate = [5e−3, 1e−3, 5e−4, 1e−4, 5e−5, 1e−5], $\lambda _1$ = [0,1,5,10,15] with or without scheduled change at every 2000 steps and batch sizes = [32, 64, 128, 256] with 50 iterations on Ascites bulk dataset as used in Scaden [9]. Other hyperparameters were fixed to the default hyperparameters of Scaden. The optimal hyperparameters were fixed for all experiments, with batch size = 64, learning rate = 1e−5, activation function = ReLU6, $\lambda _1$ according to schedule [0,15,10] at steps [0,2000,4000], and number of steps = 5000.

### Domain shift experimental setup

Using the *Ota* dataset (Additional file 1: Table S1) that contains 9852 purified samples belonging to immune cell subsets including several B cell and T cell subsets as shown in Additional file 1: Table S3, we created an experimental setup with domain shifts involving the following 4 scenarios. *20% split*: We randomly split the dataset into training (80%) and test sets (20%). *Activated 1*: We used the same split as in *20% split*. We removed certain CD4 and CD8 T cell subsets, namely, CD4 T memory, CD8 TEM, and CD8 TE from the training split while they were kept in the test set. In the test set, on the other hand, other subsets (CD4 T naive, CD8 T naive, and CD8 TCM) were removed. *Activated 2*: We followed the same procedure as in *Activated 1* except we removed certain B cell subsets, namely, B NSM, BEx, and BSM from the training set while they were kept in the test set. B naive subset was removed from the test set. Finally, for a model-based domain shift, we used DISCERN [36] to project the test set of *20% split* to the dataset simulated from *pbmc8k* and used in deconvolving PBMC bulk RNAseq. The CD4 T cell, CD8 T cell, and B cell subsets, regardless of their subtype identity, were labeled as CD4Tcells, CD8Tcells, and Bcells to allow comparisons. In each scenario, 6000 samples were simulated.

### Supplementary Information

**Additional file 1.** Supplementary tables. The file contains supplementary tables [47-74].

**Additional file 2.** Supplementary figures. The file contains supplementary figures and supplementary note [75, 76].

**Additional file 3.** Review history. The file contains the peer review history.

## Data Availability

The datasets analyzed in this work are publicly available. Summary of the datasets are provided in Additional file 1: Tables S1 (bulk) and S2 (single-cell). PBMC single-cell RNA seq was obtained from 10x Genomics [37]. Single-cell RNA-seq dataset of the brain was obtained from Allen Brain Map [38]. Single-cell RNA-seq datasets from kidneys and pancreas can be accessed using Gene Expression Omnibus using corresponding accession codes: GSE157079 (*Miao*) and GSE107585 (*Park*), GSE81608 (*Xin*), GSE84133 (*Baron*). The raw single-cell data for *Segertolpe* is available at ArrayExpress (EBI) with accession code E-MTAB-5061. Cross-tissue Immune Cell Atlas (ICA) is available from [39]. Bulk RNA-seq datasets titled *Monaco I*, *Monaco II*, *GSE120502*, *GSE107572*, *GSE50244*, and *GSE81492* are available from Gene Expression Omnibus [40] with following accession codes: GSE107011, GSE106898, GSE120502, GSE107572, GSE50244, and GSE81492. Bulk RNA-seq dataset *SDY67* was obtained from data resource provided in [9]. The original source for *SDY67* is ImmPort with accession code SDY67. *ROSMAP* cohort dataset is available from Synapse [41] with accession code syn3219045. The preprocessed data was obtained from [42]. The bulk proteomics data of post-mortem human brain samples was obtained from MAXOMOD consortium [43]. Allen brain reference with cortex annotations was obtained from [44].

## Code availability

DISSECT is implemented in Python using tensorflow and keras (both versions 2.7.0) frameworks. The code is available at GitHub [45] with installation and usage instructions and has been deposited to Zenodo [46] under MIT license.
