# Supplementary material for: DISSECT: deep semi-supervised consistency regularization for accurate cell type fraction and gene expression estimation
Source: Genome Biol. 2024 Apr 30;25:112. doi: 10.1186/s13059-024-03251-5 (PMC11061925; doi:10.1186/s13059-024-03251-5)
Supplement: Supplementary file 1 — Additional file 1. Supplementary tables. The file contains supplementary tables [47-74]. [file 13059_2024_3251_MOESM1_ESM.pdf]

DISSECT: deep semi-supervised consistency regularization for accurate cell type fraction and gene expression estimation
# – Supplementary tables –

Robin Khatri, Pierre Machart, Stefan Bonn*

Institute of Medical Systems Biology, Center for Molecular Neurobiology
Center for Biomedical AI
University Medical Center Hamburg-Eppendorf, Hamburg, Germany

*To whom correspondence should be addressed; E-mail address: sbonn@uke.de.

Datasets

Table S1: Details on bulk datasets used to evaluate deconvolution methods. For six datasets, the ground truth proportions were available while for others, relationship with the biological phenotypes was considered. Biological hypotheses based on literature serve as proxy ground truths. These are listed in ”Biological hypothesis based on literature”.

Tissue	Dataset	# samples	# Type	Flow cytometry	Biological hypothesis based on literature	Original Source
PBMC	SDY67	12	RNA-seq	Yes	-	[47]
PBMC	Monaco I	12	RNA-seq	Yes	-	[48]
PBMC	Monaco II	164	Microarray	Yes	-	[48]
PBMC	GSE65133	20	Microarray	Yes	-	[49]
PBMC	GSE107572	9	RNA-seq	Yes	-	[50]
PBMC	GSE120502	250	RNA-seq	Yes	-	[51]
PBMC	Ota	9852	RNA-seq	-	-	[52]
Pancreas	GSE50244	89 (77 with information on hemoglobic 1C levels)	RNA-seq	No	Fraction of beta cells are negatively associated with severity of type 2 diabetes indicated by hemoglobin A1c (hba1C) level [53]-[55].	[56]
Kidney	GSE81492	10	RNA-seq	No	Tubule cells diminish with chronic kidney disease (CKD) [57]-[59].	[60]
Brain	ROSMAP	508 (463 with corresponding annotation of Braak stages)	RNA-seq	No	1. Neurodegeneration with advanced Braak stage [61]-[63], and 2. Between 3:1 and 9:1 ratio of excitatory and inhibitory neurons [64]-[68].	[69]
Brain	PFC proteomics	50	Mass spec.	No	Between 3:1 and 9:1 ratio of excitatory and inhibitory neurons [64]-[68].	[43]
Lymph node	Lymph node	4,035 spots	10x Visium	No	Identification of germinal centers (GC) by co-localization of GC associated cell types	10x Genomics
Brain	Anterior sagittal mouse brain	2,695 spots	10x Visium	No	Identification of excitatory neuronal layers	10x Genomics

Single-cell datasets

Table S2: Single cell datasets used as reference. To deconvolve PBMC datasets in Table S1, single-cell datasets from the corresponding tissues were considered. We used the *PBMC8k* as a reference single-cell dataset from a healthy donor for all methods considered here. To maintain same genes between the single-cell data and bulk RNA-seq, we subset both datasets over common gene-set. For the multi-sample setting to use with MuSiC, we considered *Immune Cell Atlas (ICA)*. The atlas was restricted to blood to match the bulk tissue with donor: 621B (103 cells), 637C (760 cells), A35 (1,368 cells), A36 (3,124 cells), D496 (9,065 cells), D503 (12,208 cells). Cell types that were present in less than 5 samples were dropped. All PBMC datasets were annotated with B cells, CD4 T cells, CD8 T cells, Monocytes and NK cells while the remaining cells were mixed to form an unknown cluster. To deconvolve pancreas, kidney, brain and lymph node samples in Table S1, single-cell dataset from the corresponding tissues were considered.

**Tissue**	**Dataset**	**# cells**	**Multi-sample**	**Original source**
PBMC	PBMC8k	8,381	no	10x Genomics (8k PBMCs from a Healthy Donor)
PBMC	PBMC6k	5,419	no	10x Genomics (6k PBMCs from a Healthy Donor)
PBMC	DonorA	2,900	no	10x Genomics (Frozen PBMCs Donor A)
PBMC	DonorC	9,519	no	10x Genomics (Frozen PBMCs Donor C)
PBMC	Immune Cell Atlas (ICA)	26,628	yes	[15]
Pancreas	Baron	8,569	yes	[70]
Pancreas	Segerstolpe	3,514	yes	[71]
Pancreas	Xin	1,492	yes	[72]
Kidney	Park	43,745	yes	[73]
Kidney	Miao	16,887	no (only adult)	[74]
Brain	Allen Brain Atlas	49,418	yes	[38]
Lymph node, spleen, tonsil	lymph node reference	73,620	yes	[29]

Table S3: Pearson correlation coefficient (r) between estimates from different methods and flow cytometry for granular cell type fractions in *Monaco I*.

**Dataset**	**MuSiC**	**CSx**	**Scaden**	**TAPE-O**	**TAPE-A**	**Linear MLPs**	**DISSECT**
B Ex	nan	**0.43**	0.18	0.22	0.030	0.10	0.32
B NSM	nan	-0.08	**0.12**	0.10	-0.15	-0.22	0.09
B Naive	nan	0.95	0.87	0.8	0.43	0.71	**0.96**
B SM	**0.85**	nan	0.57	0.45	0.15	0.26	0.63
Monocytes C	0.30	0.29	**0.63**	0.57	0.52	0.11	0.62
Monocytes I	0.41	0.36	0.90	0.87	0.81	0.54	**0.93**
Monocytes NC	0.25	0.09	0.31	0.35	0.48	0.19	**0.66**
NK	0.80	**0.82**	0.58	0.59	0.65	0.49	**0.82**
Neutrophils LD	0.2	nan	**0.89**	0.48	0.57	0.03	0.56
Plasmablasts	0.62	0.85	0.86	0.65	0.66	0.42	**0.92**
CD4 T Naive	0.66	0.47	0.68	0.70	0.34	0.14	**0.76**
CD4 T Memory	**0.47**	-0.15	0.27	0.27	0.12	0.08	0.24
CD8 T Naive	0.52	**0.7**	0.36	0.38	0.32	0.27	0.49
CD8 T CM	nan	-0.65	0.19	0.13	**0.21**	0.01	0.12
CD8 T EM	nan	nan	0.02	0.47	0.45	0.11	**0.62**
CD8 T TE	0.25	**0.9**	0.28	0.35	0.36	0.35	0.86
mDC	nan	0.46	0.47	0.39	0.40	0.05	**0.68**
pDC	0.55	**0.57**	0.19	0.42	0.31	0.3	0.55
**Average**	0.49	0.40	0.46	0.45	0.37	0.22	**0.60**

Table S4: *rmse* between estimates from different methods and flow cytometry for granular cell type fractions in *Monaco I*.

**Dataset**	**MuSiC**	**CSx**	**Scaden**	**TAPE-O**	**TAPE-A**	**Linear MLPs**	**DISSECT**
B Ex	**0.01**	0.05	0.04	0.04	**0.01**	0.02	0.02
B NSM	0.02	**0.01**	0.02	0.03	0.05	0.04	0.02
B Naive	**0.01**	0.03	0.03	0.03	0.04	0.06	0.03
B SM	0.05	**0.01**	**0.01**	0.02	0.02	0.03	**0.01**
Monocytes C	0.05	**0.02**	0.04	**0.02**	**0.02**	0.06	0.06
Monocytes I	0.12	0.15	**0.03**	0.06	0.10	0.12	0.04
Monocytes NC	0.20	0.09	**0.02**	0.07	0.05	0.10	**0.02**
NK	**0.05**	0.08	0.08	0.11	0.11	0.05	0.08
Neutrophils LD	0.02	0.03	**0.01**	**0.01**	**0.01**	0.01	0.02
Plasmablasts	**0.01**	**0.01**	0.02	**0.01**	0.04	0.01	0.04
CD4 T Naive	**0.02**	0.03	0.05	0.05	**0.02**	0.02	0.05
CD4 T Memory	0.10	0.07	**0.03**	0.12	0.15	0.21	**0.03**
CD8 T Naive	0.21	0.07	**0.04**	0.05	0.05	0.01	**0.04**
CD8 T CM	**0.01**	0.08	0.02	0.05	0.12	0.01	0.03
CD8 T EM	0.02	**0.01**	0.02	0.02	**0.01**	0.03	0.02
CD8 T TE	**0.01**	0.07	0.09	0.08	0.11	0.16	0.08
mDC	**0.01**	0.04	0.04	0.08	0.09	0.03	0.03
pDC	0.02	**0.00**	0.02	0.01	0.01	0.01	0.02
**Average**	0.06	0.05	**0.03**	0.05	0.06	0.05	0.04

Table S5: Average performance over five random experiments for SDY67 (Table S1.) Each column indicates the additional part.

Metric	Linear MLP	Activations	KL Divergence	KL Divergence + Consistency
r	0.51 ± 0.018	0.55 ± 0.016	0.54 ± 0.006	0.63 ± 0.005
rmse	0.13 ± 0.008	0.13 ± 0.006	0.11 ± 0.004	0.09 ± 0.002
