# Supplementary material for: DISSECT: deep semi-supervised consistency regularization for accurate cell type fraction and gene expression estimation
Source: Genome Biol. 2024 Apr 30;25:112. doi: 10.1186/s13059-024-03251-5 (PMC11061925; doi:10.1186/s13059-024-03251-5)
Supplement: Supplementary file 2 — Additional file 2. Supplementary figures. The file contains supplementary figures and supplementary note [75, 76]. [file 13059_2024_3251_MOESM2_ESM.pdf]

DISSECT: deep semi-supervised consistency regularization for accurate cell type fraction and gene expression estimation
# – Supplementary figures and Supplementary Note –

Robin Khatri, Pierre Machart, Stefan Bonn*

Institute of Medical Systems Biology, Center for Molecular Neurobiology
Center for Biomedical AI
University Medical Center Hamburg-Eppendorf, Hamburg, Germany

*To whom correspondence should be addressed; E-mail address: sbonn@uke.de.

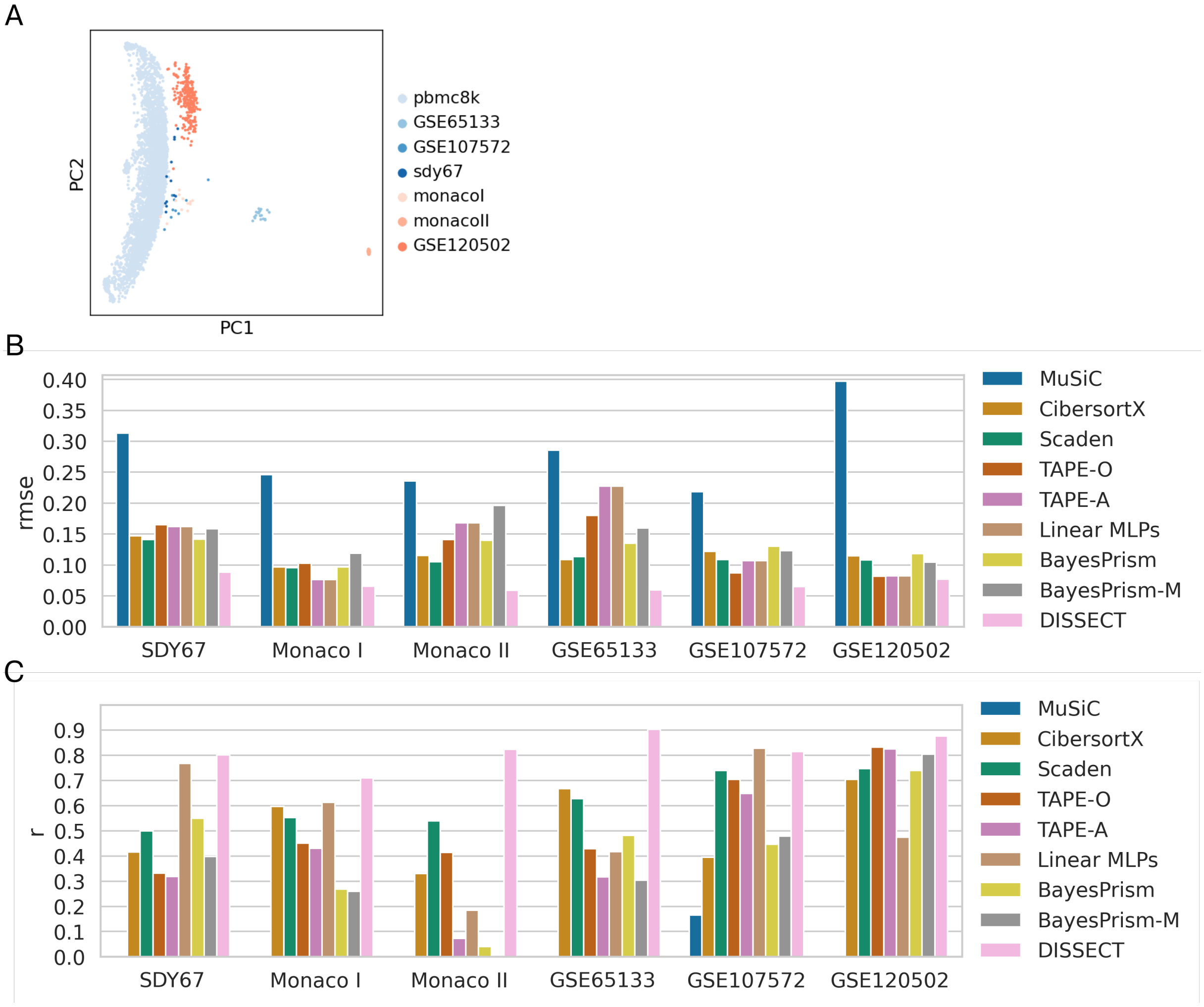

Fig. S1: **A.** PC (Principal component) embeddings of simulated and real PBMC datasets computed using the union of the top 2,000 highly variable genes per dataset. **B.** Overall Pearson's correlation (*r*) and **C.** root-mean-squared-error (*rmse*) for each of the dataset. Datasets are listed on x-axis.

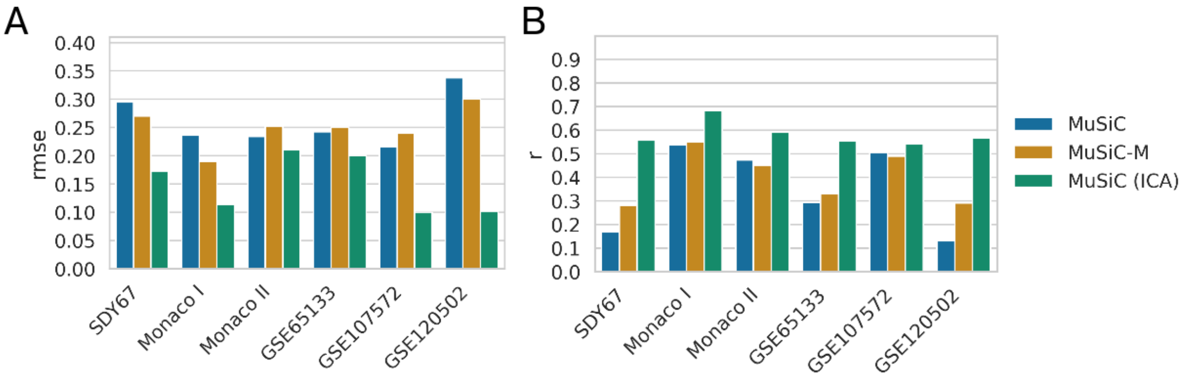

Fig. S2: Comparison of MuSiC (Fig. 3D), MuSiC with marker genes (MuSiC-M) and MuSiC with blood data from Immune Cell Atlas *ICA* (MuSiC-ICA). **A**. root mean-squared-error (*rmse*) and **B**. Pearson's correlation (*r*).

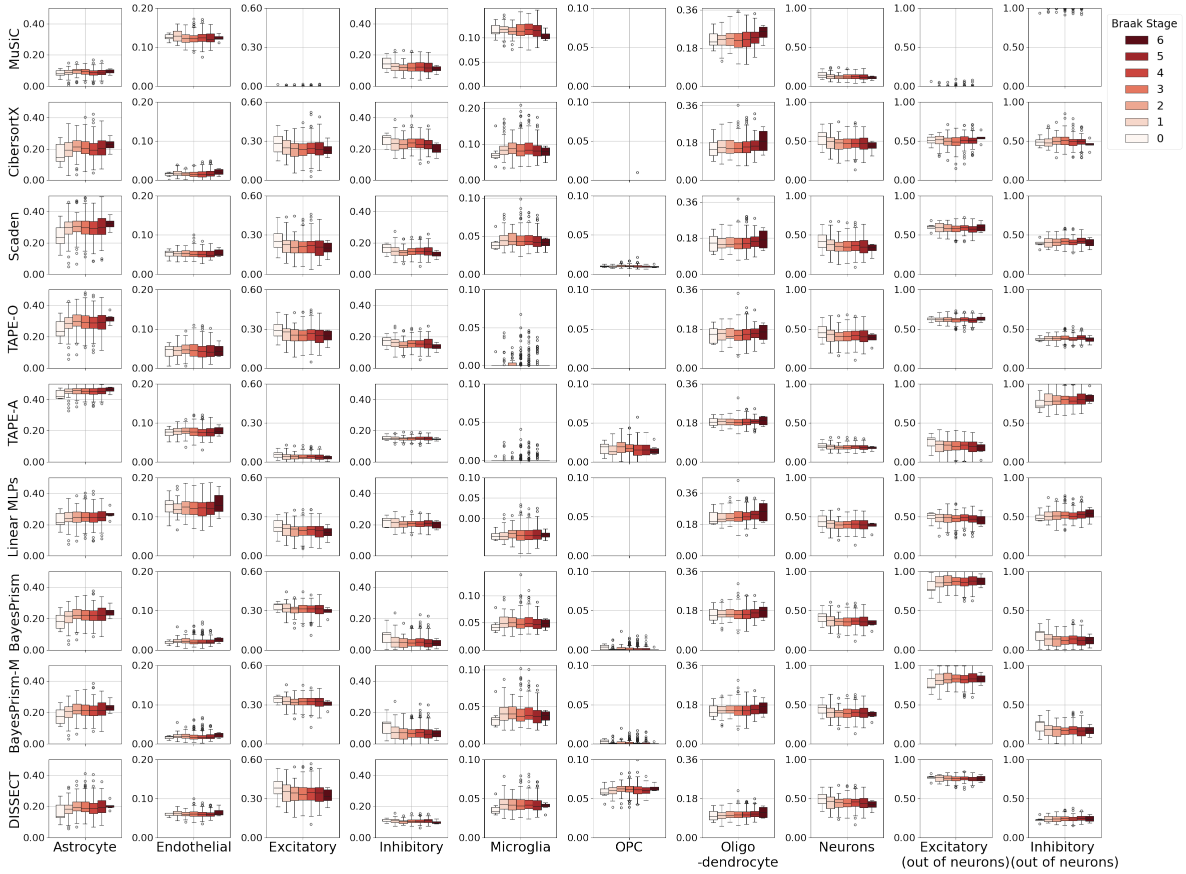

Fig. S3: Estimated cell type proportions on *ROSMAP* separated by Braak Stage. Rows indicate methods, columns indicate cell type.

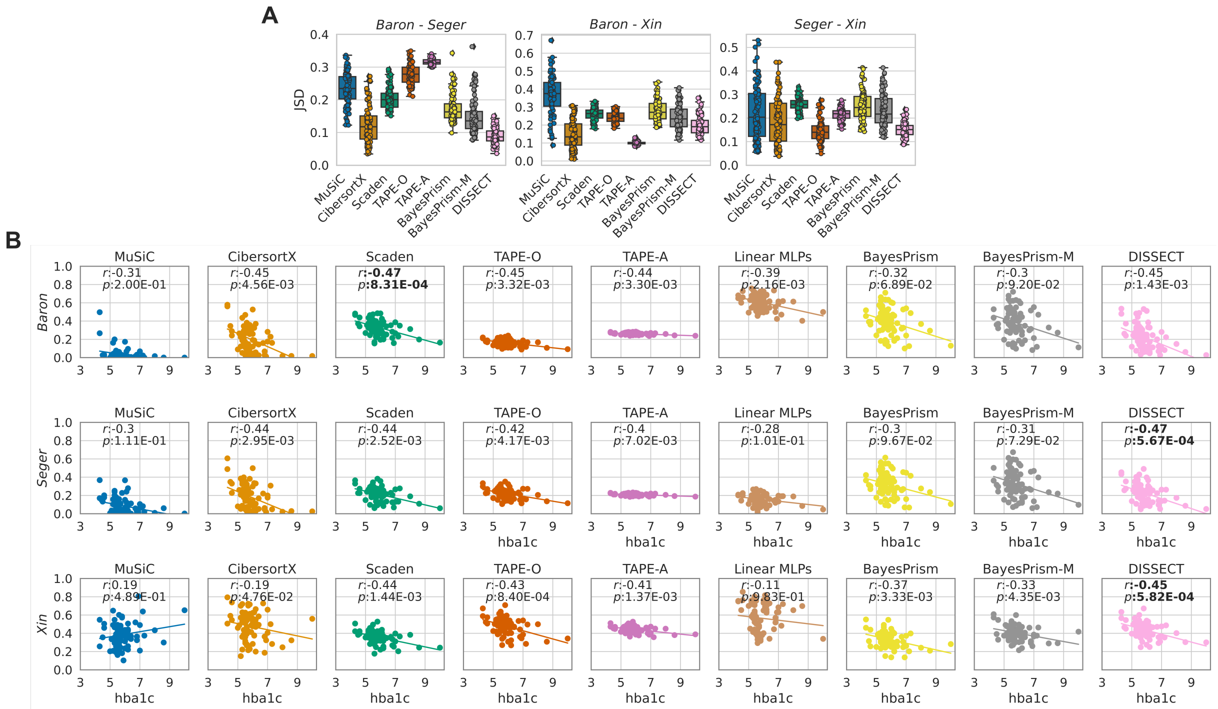

Fig. S4: **A**. Box-plots showing *JSD*s between predicted proportions from *Pancreas* using different single-cell references. Each plot shows *JSD*s between two references. From left to right: *Baron* and *Seger*, *Baron* and *Xin*, and *Seger* and *Xin*. **B**. Associations between predicted beta proportions and hba1c levels assessed through multiple linear regression with hba1c as dependent variable and beta estimates, age, BMI and gender as independent variables. *P*-values correspond to 2-tailed Student's t-test for significance of coefficients for beta estimates. *r* is the Pearson correlation coefficient between beta estimates and hba1c. Each column indicates a method and each row indicates a reference.

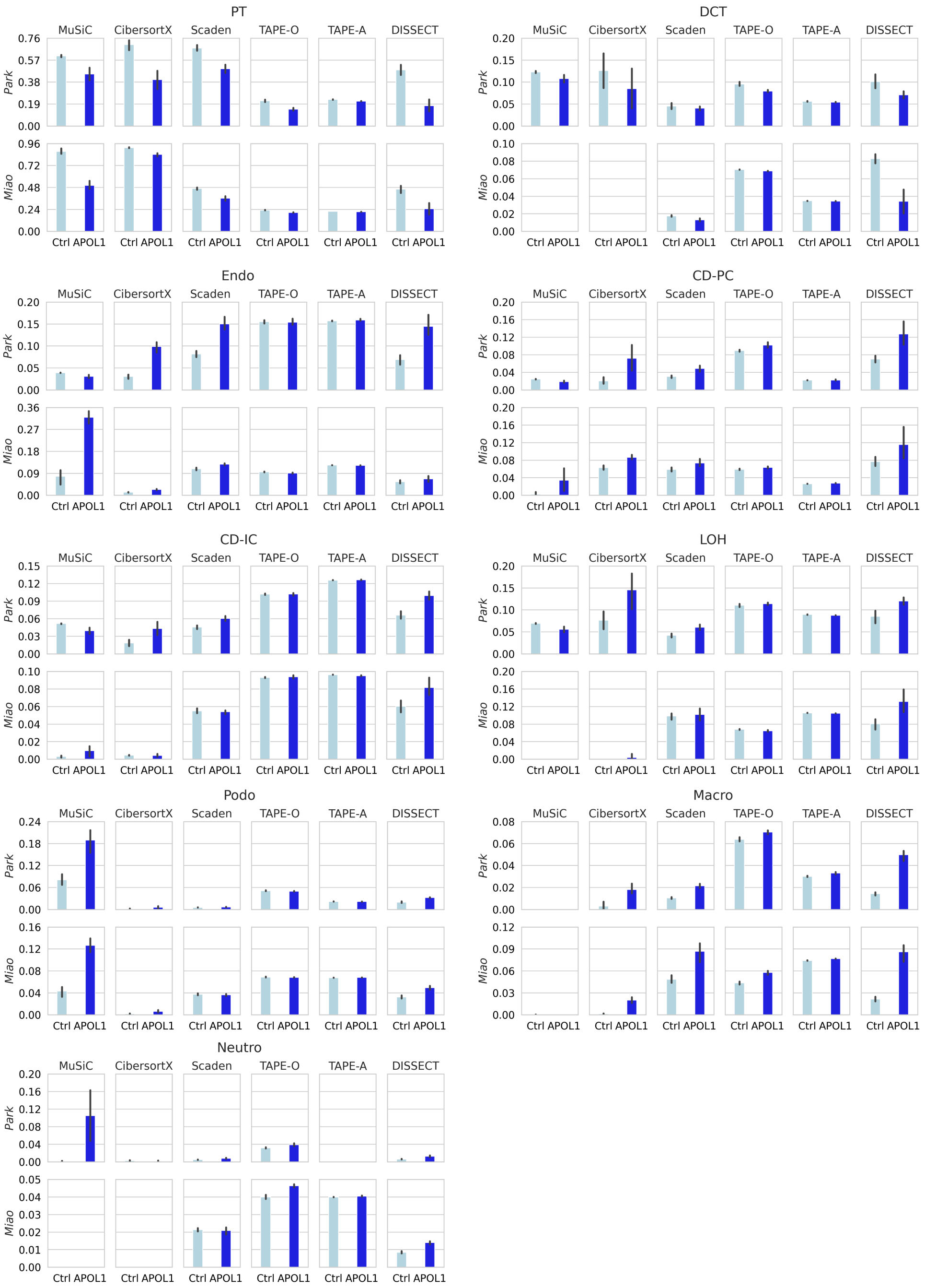

Fig. S5: The figure extends Fig. 3D to include all methods and cell type. From left to right and top to bottom: Proximal tubule (PT), ductal convoluted tubules (DCT), endothelial cells (Endo), collecting duct principal cells (CD-PC), collecting duct intercalated cells (CD-IC), loop of henle (LOH), podocytes (Podo), macrophages (Macro) and neutrophils (Neutro). Each row indicates a reference (*Miao, Park*).

Supplementary Note

To exemplify the applicability of DISSECT on other data types. We aimed to deconvolve spatial transcriptomics datasets. The 10x Genomics Visium^{TM} platform, for instance, delivers spatial gene expression information with a spot diameter of 55 μm. This resolution is not enough to capture single cells and spot-based gene expression on Visium^{TM} is therefore a mixture of its constituent cells. In this section we measure DISSECT's deconvolution performance on Visium^{TM} ST data.

We performed deconvolution on two ST samples obtained from 10x Genomics website corresponding to Lymph node and brain and compared against RCTD and Lymph node. Both datasets are accompanied by H&E images of the underlying tissue.

Anterior sagittal mouse brain - Brain is a highly structured organ with information about the structures of neurons in the cortex. We utilized the reference data from Allen Brain Atlas, as previously used in deconvolution of *ROSMAP* data. Using the ST adapted simulations, we computed proportions of different cell types, including different layers of neurons and visualized them on top of a corresponding hematoxylin and eosin (H&E) stained image (Fig. S6A). DISSECT faithfully captured the spatial layering of the cortical areas of the brain, as well as known 'hot-spots' of neurons, oligodendrocytes, astrocytes, and inhibitory neurons such as somatostatin- and parvalbumin-positive neurons.

To identify cortical layers, we performed louvain clustering (resolution 1) on the estimated cell type fractions per spot, and labelled the clusters enriched for different layers of neurons. Layers L2/3 IT, L4, L5 IT, L5 PT, L6 CT, L6 IT, and L6b were mapped respectively to clusters 1, 7, 12, 8, 3, 14 and 15 (Fig. S6B,C). The identified cortical layers corresponded with the cortical layers annotated in the Allen Reference Atlas (Fig. S6D). We applied RCTD, C2L, SONAR and CARD using the same setting. Compared to RCTD and C2L, DISSECT achieves better separation of excitatory neuronal layers. CARD slightly outperforms DISSECT in this task with a 0.02 increase in schilloute scores. The quantification was made using silhouette score with euclidean metric. (Fig. S6E).

Lymph node - Next, we evaluated DISSECT on the spatial deconvolution of lymph node tissue. Lymph nodes consist of various immune cell subsets and localized germinal centers (GCs). We used a lymph node single-cell reference and used the manually annotated germinal centers that were provided with the study as ground truth [29]. To verify whether DISSECT estimated and localized cell fractions per spot correctly, we visualized GC-related cell types, namely Cycling B cells, Germinal center B cells and follicular dendritic cells (FDCs) (Fig. S7A). DISSECT also identified T cells associated regions in and around these GCs (Fig. S7A). To obtain binary GC predictions to compare with the ground truth GC annotations, we computed louvain clusters (resolution 1) on the cell type proportions, and labelled spots with cluster 1 as GCs based on enrichment of GC associated cell types (Fig. S7B). Since the number of GC spots (378) is considerably lower than non-GC spots (3,657), balanced accuracy as implemented in Scikit-learn was used to account for this imbalance [75]-[76]. Comparison with the ground truth revealed a balanced accuracy of 0.94, indicating that DISSECT deconvolved GCs with high accuracy and

on par performance with C2L and RCTD (Fig. S7C). For CARD and SONAR, the balanced accuracy were 0.93 and 0.91 respectively.

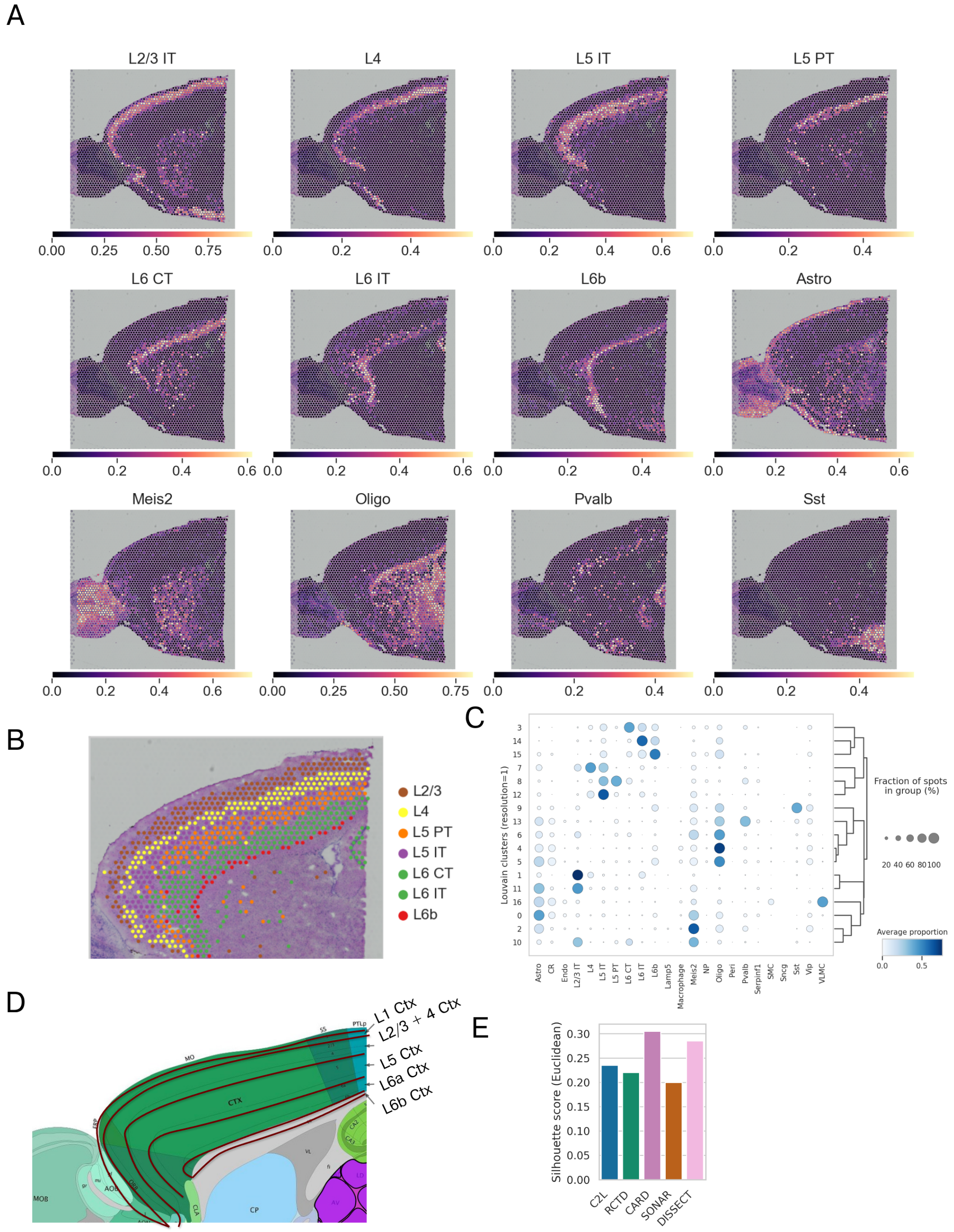

Fig. S6. **A**. Estimated cell type proportions from mouse brain tissue visualized over H&E image of the corresponding tissue. L2/3 - L6b indicate different layers of neurons. *Astro:* Astrocytes, *Oligo:* Oligodendrocytes. **B**. Estimated cortical layers using enrichment of cell type proportions in louvain clusters presented in **C**. **D**. Annotations of cortical layers from Allen Brain Reference obtained from [38]. For visibility, cortex boundaries were highlighted. **E**. Shilloute scores for C2L, RCTD, SONAR, CARD and DISSECT.

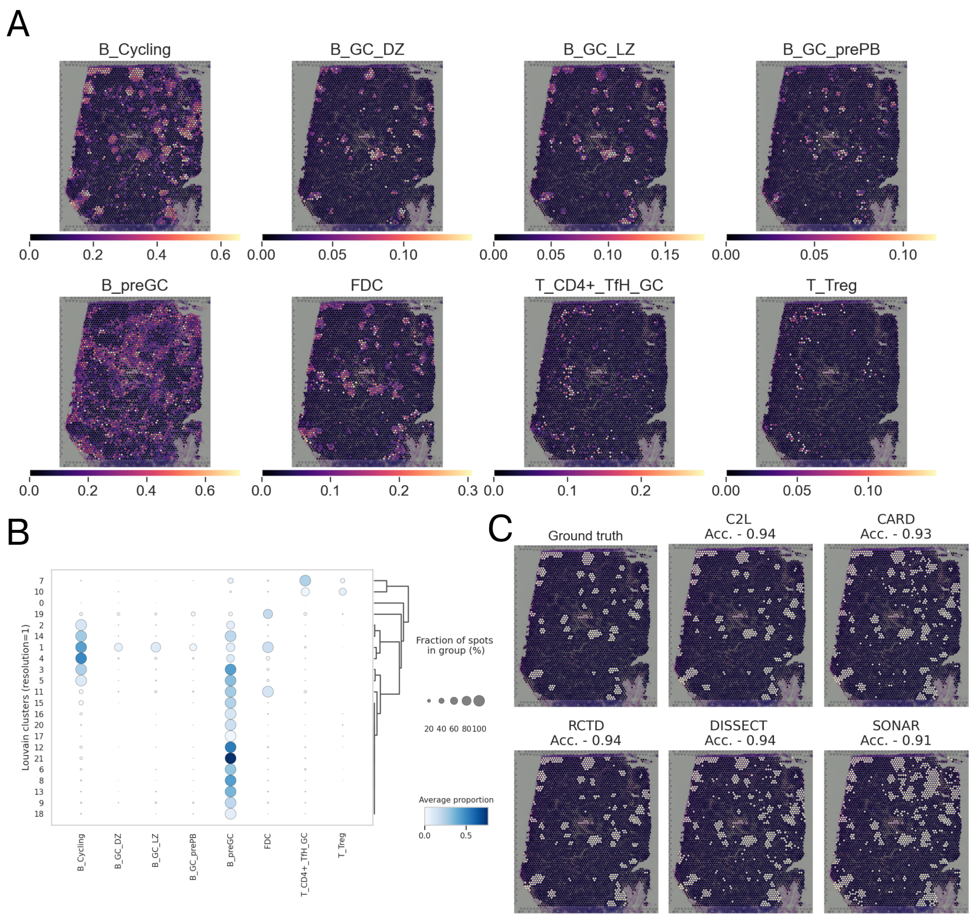

Fig. S7: **A**. Estimated cell type proportions visualized over H&E image of the corresponding lymph node tissue. *B_cycling*: Cycling B cells, *B_DC_DZ*: Dark zone germinal center B cells, *B_GC_LZ*: Light zone germinal center B cells, *B_GC_PrePB*: germinal center Pre-plasmablast/plasma cells, *B_preGC*: pre-germinal center B cells, *FDC*: Follicular dendritic cells, *T_CD4$^+$TfH_GC*: Germinal center follicular helper CD4$^+$ T cells, *T_Treg*: Regulatory T cells. **B**. Louvain clustering on cell type proportions. y-axis lists cluster numbers and x-axis lists cell types. **C** Comparison of identified clusters, from left to right and top to bottom: Ground truth GC-spots, predicted annotations for C2L, RCTD, DISSECT, CARD and SONAR.

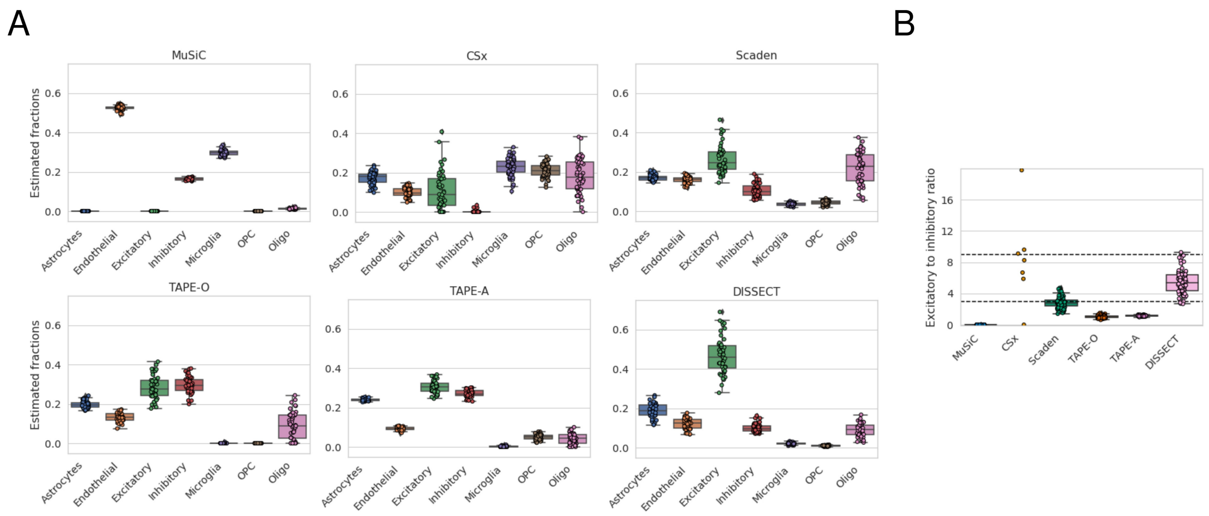

Fig. S8: **A**. Boxplots showing estimated cell type proportions from human proteomics samples. Title of each plot is indicated at the top of the plot and x-axis list cell types. **B**. Boxplots showing predicted excitatory to inhibitory neuron ratios for each method for the proteome samples. Expected ratios lie between 3:1 and 9:1 as indicated by dashed lines. To make the plot discernible possible, in **B**, the y-axis was limited to a value of a maximum value of 17.

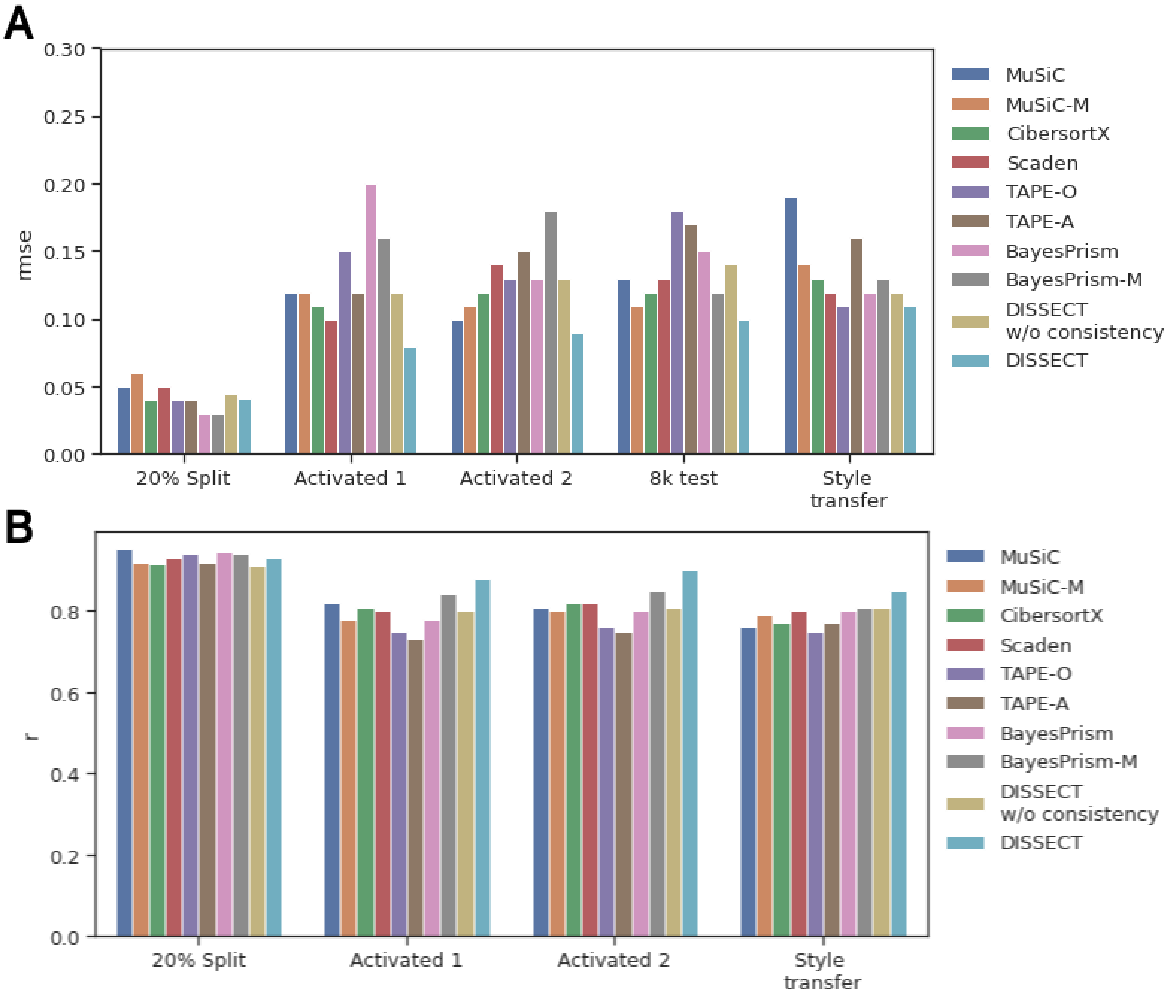

Fig. S9: Performance of deconvolution algorithms in estimating cell types fractions under domain shift.
