# Supplementary material for: DISSECT: deep semi-supervised consistency regularization for accurate cell type fraction and gene expression estimation
Source: Genome Biol. 2024 Apr 30;25:112. doi: 10.1186/s13059-024-03251-5 (PMC11061925; doi:10.1186/s13059-024-03251-5)
Supplement: Supplementary file 3 — Additional file 3. Review history. The file contains the peer review history. [file 13059_2024_3251_MOESM3_ESM.docx]

**1^st^ round**

**Reviewer 1**

Khatri, Machart, and Bonn describe a new method for performing deconvolution of high-throughput biological data where the main goal is to estimate cell fractions in each sample. This method is called DISSECT and it is semi-supervised in the sense that you do need to provide a reference input dataset with the different cell types labeled as such. DISSECT is versatile as it can be applied to deconvolve bulk RNA-seq data using scRNA-seq as the input reference, but it can also work with proteomic data as well as spatially-resolved transcriptomics data (tested namely using Visium data by 10x Genomics). Overall the authors showed that DISSECT slightly underperforms compared to other methods, to outperforming them. Thus, DISSECT is at worse about equal to other methods, and at its best, it does provide more accurate results. On average, it performs better on the different datasets used to evaluate and showcase DISSECT. Questions remain on the limits of DISSECT in terms of rare cell types or similar cell types. The overall software documentation is minimal and would greatly benefit from a significant effort to improve it, for example, by adding tutorials with easy to access data that users can follow step by step. Given that in several cases DISSECT provides comparable results to other existing methods that have extensive software documentation, I believe that DISSECT's authors will face an uphill battle to convince users from those tools to switch over, unless efforts to improve DISSECT's documentation are carried out. DISSECT can also be used to estimate cell type specific expression levels, though the comparison is mainly limited against TAPE-A. The software documentation for DISSECT claims that it can benefit from using GPUs, but not much information is provided there or in the manuscript about what are the speed gains, how to do so, or any other computational requirements.


* Page 5: define JSD and RMSE acronyms since this if the first time you mention them.
* How did you choose the marker genes? From our own work, we know that this is very important for tools like MuSiC.
* Unless I missed it, I don't think that you have made your analysis code public. Doing so would be very useful.
* Page 27 line 12. "we modify simulation procedure". Should it be: "we modified the simulation procedure"?
* It seems that in order to apply DISSECT to Visium data, DISSECT would be able to provide the estimated cell fractions per spot (summing to 1), and then users would need to use an external method to estimate the number of cells for each spot. With that, they could multiply the two to estimate the number of cells per spot for each cell type. Is this correct? If so, mentioning these steps would be useful for DISSECT users looking to apply it to Visium data.
* While https://github.com/robinredX/DISSECT provides steps for running DISSECT, the information provided is very sparse. It would be useful to show how to use DISSECT with some of the public datasets described in the paper. Doing so will enable users to compare their objects and outputs at different parts and contrast them with those on the tutorial.
* The information at https://github.com/robinredX/DISSECT on how to use a GPU is nearly non-existent. Please provide more information on how to do so.
* Please deposit https://github.com/robinredX/DISSECT to Zenodo, Figshare, or some other location that will provide a DOI and a permanent home for the code (code can always be deleted from GitHub). See https://docs.github.com/en/repositories/archiving-a-github-repository/referencing-and-citing-content for more details for integrating GitHub and Zenodo. You can then cite the Zenodo (Figshare, etc) DOI on your paper next to the GitHub link for the code repository.
* "Especially for hard deconvolution tasks, such as samples with many similar cell types and very scarce cell populations, an increase in deconvolution performance is warranted." I think this sentence needs to be expanded (likely a few more sentences). Currently there is no guide for users to know what is the threshold for defining "very scarse cell populations": is it below 10%? 5%? 1%? Similarly, there are no guidelines for defining "similar cell types". You could mention whether DISSECT worked for X and Y similar cell types in dataset 1, but for A and B similar cell types in dataset 2 it didn't. That would give readers the background on which datasets were used for defining these practical guidelines. Otherwise, right now, users are left blind and need to do full explorations themselves to find these thresholds. That is a lot of work and will turn users away from DISSECT.
* Note that https://doi.org/10.1101/2023.03.13.532468 evaluated other deconvolution methods that estimate the cell type specific expression levels. They found bMIND to be the best performer. Overall, it seems to me that it would be useful to compare DISSECT against other tools than just TAPE-A.
* The caption for Figure S5 overlaps the page number. This might be out of your control. It also happens with other pages in the supplement.

**Reviewer 2**

In this paper the authors proposed two semi-supervised deep learning models for cell type deconvolution and cell type expression signature estimation, named DISSECT. The MLP model is used for deconvolute cell type fractions. The authors have benchmarked this model on both simulation and real bulk datasets. They also show that their model is possible to be extended to proteomic data and spatial transcriptomic data. Their second model applies a VAE framework to estimate or update cell type specific gene expression. This model is only tested on simulation data. The authors claim that the consistency regularization design in both models can account for the domain shift between the training and target data.

Single-cell guided bulk deconvolution is a classic problem, and has been discussed for many years. It is still an interesting topic but may not be as catchy as in past years. While the use of semi-supervised deep learning framework and the application of consistency regularization in DISSECT can be a valuable addition to this field, further efforts are needed to clarify the difference and advancement of this method over the others. I have the following comments：

1. I think the major issue about this manuscript is the how to clarify and elucidate the novelty and advantage of DISSECT over existing methods. For example, if you think the model's ability of domain adaptation is the major advancement, then this should be thoroughly illustrated. First, the definition of "domain" should be clearly elaborated. The words such as domain shift, domain knowledge, domain gap or domain variation may not be familiar to the general audience. Second, the authors seem only hypothesize that consistency regularization can accommodate domain shift between training and target data, but failed to show the evidence. Some visualization of the data can help illustrate existence of domain gap. Comparisons of performance on data with and without domain gap are also necessary.

2. The necessity of estimation of type specific expression profile from bulk should be explained. If there is enough single-cell data, I don't see why it is needed to make the double step estimation for cell type-specific expression. The result part on Page 19 Line 42 only proves the applicability of the second model. However, it does not convince me of the need for such a model if no real data application is exemplified.

3. Regarding the model traning on P.9 L.21, how do you loop over true samples? Or do you just randomly draw a true sample at each step? Also, how batches were generated? Do you divide samples into mini-batches in advance? At each step, you use a true sample and pair with it a simulated pseudo-sample?

4. In the second model, do treat f as known which is a completely trained mapping from model 1? Or can f be also trained in this model?

5. In your simulations studies, how many bulk samples are simulated for each experiment?

6. BayesPrism is another methods that perform deconvolution as well as cell type specific expression estimation, which should be compared.

7. In spatial transcriptomics data the number of samples (spots) may approach 5000, how do you adjust the number of steps? There are methods designed with specific for ST data which utilize spatial information, e.g. CARD, SONAR, SD2. How do DISSECT compared to these methods?

8. The evaluation in section "Estimation of cell type-specific gene expression" should be clarified. It is not clear how sample-wise and gene-wise correlations are calculated.

9. It is not clear how ablation study was performed (P.14 L.41)

Minor comments:
10. Can you elaborate or at least briefly show how the grid search was performed in choosing the optimal activation function. Do you have to re-tune the hyper-parameters such as \lambda_1, \lambda_2, \beta^VAE and the learning rate each time when applying DISSECT to a new dataset?
11. In the sentence on P.5 L.41, "… consistency-based regularization penalizing the non-linearity of mixtures of real and simulated samples…", can you explain what is the non-linearity of mixtures of real and simulated samples?
12. The author proposed two models but only gave one name, DISSECT. I would be more convenient if each model is assigned with a sub-name.
13. P.10 L.14, CSx should be presented in full name for the first appearance in the MS.
14. There are some typos：
P.10 L.41 should it be "-" instead of "+" for the last term?
P.11 L.48 … two evaluate…-> to evaluate
P.22 L.37 there are 2 "y" in eqn7

**Authors’ response**

Reviewer 1:

We thank the reviewer for the many very detailed and constructive criticisms and suggestions that have led to both scientific and structural improvements of our work. We particularly appreciate the reviewer’s questions regarding limits of DISSECT on rare and similar cell types, suggestions for improvement in the documentation of DISSECT, and other thoughtful comments that have greatly benefited the manuscript and software.

(1) Page 5: define JSD and RMSE acronyms since this if the first time you mention them.

We have now introduced the abbreviations in the Background section (page 3), where they are first mentioned.

(2) How did you choose the marker genes? From our own work, we know that this is very important for tools like MuSiC.

We thank the reviewer for this comment. We agree that marker gene selection is a crucial step for a deconvolution algorithm.

Among the methods that we have now compared in our work for deconvolution ,

1. CibersortX computes a set of marker genes to create a reference cell type specific signature matrix. We have added the following information in the description of CibersortX in page 23. To construct the signature matrix, we used the following hyperparameters for CibersortX as recommended by the authors: kappa = 999, q- value = 0.01 and number of genes within a range of 300 and 500. The quantile normalization was also disabled.

2. MuSiC assigns a weight to each gene instead of computing marker genes. “Because it is a linear regression-based method, genes showing less cross-cell type variations will have low leverage, thus having less influence on the regression, whereas the most influential genes are those with high weight and high leverage. To illustrate this point, we also performed benchmarking experiments to show that applying MuSiC using all genes gives more accurate results than applying MuSiC using pre-selected marker genes, thus demonstrating that MuSiC’s weighting scheme makes marker gene pre-selection unnecessary” (Wang et al., 2019).

Another variable that affects MuSiC’s performance in our hands is the usage of multi- sample references (i.e. scRNA-seq data consisting of more than two donors). In our work, we illustrated this by utilizing a large single-cell blood atlas of immune cells (n=26,628, Table S2) to deconvolve all PBMC datasets (Table S2, Rebuttal Fig 1). However, MuSiC, while improving considerably, did not show a performance at par with the best performing methods. We have added a column in Table S2 with information on whether a single-cell reference is multi-sample.

(Table S2)

Since the setting used in Wang et al., 2019 was not evaluated on bulk data with paired flow cytometry information, we evaluated MuSiC with pre-selected marker genes in these experiments. We have now evaluated MuSiC by pre-selecting marker genes as selected by CibersortX as done previously by Chu et al., 2022. To avoid confusion with the default MuSiC, we named this setting as MuSiC-M. We provide these results in the Figure S2. We observed that the performance of MuSiC-M varied between the tested datasets, with a decrease in rmse of 0.024, 0.046 and 0.038 for SDY67, Monaco I, and GSE120502, respectively, and an increase in rmse for the other three datasets evaluated. In terms of correlation, MuSiC-M provided improvements in 4 out of 6 datasets with an over 0.1 increase for SDY67 (+0.11) and GSE120502 (+0.16). We have updated the following text in the results section:

*We also evaluated MuSiC with pre-selected marker genes (MuSiC-M) that were selected by CSx. MuSiC-M showed increased performance in 4 out of 6 datasets (Fig. S2A-B). MuSiC also shows improved performance in the multi-sample setting in both rmse (Fig. S2A) and r (Fig. S2B).*

Fig 1. Comparison of MuSiC (Fig. 3D), MuSiC with marker genes (MuSiC-M) and MuSiC with blood data from the Immune Cell Atlas (MuSiC-ICA). A. root mean- squared-error (rmse) and B. Pearson's correlation (r).

We have added the following text in the description of MuSiC: *We used the default hyperparameters to execute MuSiC. For single-cell datasets with multiple donors (Table S2), we ran MuSiC with single-cell data from all available donors.*

*Chu, Tinyi, et al. "Cell type and gene expression deconvolution with BayesPrism enables Bayesian integrative analysis across bulk and single-cell RNA sequencing in oncology." Nature Cancer 3.4 (2022): 505-517.*

3. In response to the feedback we received from reviewer 2, we also integrated BayesPrism, a deconvolution method to estimate both cell type ration and gene expression estimation. BayesPrism may or may not use marker genes. Under default settings, BayesPrism does not compute marker genes. However, the authors recommend using function select.marker genes in certain scenarios (e.g. https://github.com/Danko-Lab/BayesPrism/issues/57). We have now included BayesPrism under both settings, which we refer to as BayesPrism and BayesPrism-M (BayesPrism with prior marker gene selection). We have also added a brief description of BayesPrism in the methods section.

4. The other learning-based algorithms (Scaden, TAPE-O, TAPE-A, and DISSECT) do not require computation of marker genes as they are learnt during the training.

(3) Unless I missed it, I don't think that you have made your analysis code public. Doing so would be very useful.

Thank you for this comment. We now provide the code to reproduce all figures and to run DISSECT as well other methods https://github.com/imsb-uke/deconvolution and link it to the documentation of the standalone DISSECT package at https://github.com/imsb- uke/DISSECT. (Note that CibersortX is a patented framework and requires an access token for academic work).

(4) Page 27 line 12. "we modify simulation procedure". Should it be: "we modified the simulation procedure"?

We have corrected it in the revised manuscript.

(5) It seems that in order to apply DISSECT to Visium data, DISSECT would be able to provide the estimated cell fractions per spot (summing to 1), and then users would need to use an external method to estimate the number of cells for each spot. With that, they could multiply the two to estimate the number of cells per spot for each cell type. Is this correct? If so, mentioning these steps would be useful for DISSECT users looking to apply it to Visium data.

The reviewer is correct that DISSECT estimates proportions, which could be used to estimate the number of cells per cell type using existing methods. This problem is closely linked to mapping single cells to spots based on deconvolved proportions, which can be achieved using CytoSpace (Vahid et al., 2022), or instance. We thank the reviewer for this comment and we agree that this provides useful information to end-users. We have now added the following text in the Supplementary text.

For the spatial transcriptomics data, DISSECT estimates cell type fractions per spot, which are constrained to sum to 1. To be able to estimate the number of cells per cell type for each spot, and to map single cells, DISSECT estimates can be used as a prior for algorithms such as CytoSpace [33]. CytoSpace infers both the number of cells in each spot and solves an optimization problem to map single cells to their spatial locations. To estimate only the number of cells per cell type for each spot, the total number of cells as estimated by CytoSpace can be multiplied with the output of DISSECT.

(6) While https://github.com/robinredX/DISSECT provides steps for running DISSECT, the information provided is very sparse. It would be useful to show how to use DISSECT with some of the public datasets described in the paper. Doing so will enable users to compare their objects and outputs at different parts and contrast them with those on the tutorial. * The information at https://github.com/robinredX/DISSECT on how to use a GPU is nearly non- existent. Please provide more information on how to do so.

and

(7) Please deposit https://github.com/robinredX/DISSECT to Zenodo, Figshare, or some other location that will provide a DOI and a permanent home for the code (code can always be deleted from GitHub). See https://docs.github.com/en/repositories/archiving-a- github-repository/referencing-and-citing-content for more details for integrating GitHub and Zenodo. You can then cite the Zenodo (Figshare, etc) DOI on your paper next to the GitHub link for the code repository.

We have taken steps to improve the documentation of DISSECT’s setup and usage. For a long term support, we have maintained the repository now at the institution’s namespace: https://github.com/imsb-uke/DISSECT, which includes better documentation, tutorials as well as easily installable DISSECT package that can be run from Python functions rather than command line. We have also added additional information on how to use GPUs to the documentation. Furthermore, we have added tutorials detailing the required input data formats and how the results are structured.

The code has been deposited to Zenodo and is available with the following link and DOI: https://zenodo.org/doi/10.5281/zenodo.10570403.

We have updated the text in the “Availability of data and materials” section: *DISSECT is implemented in Python using tensorflow and keras (both versions 2.7.0) frameworks. The code is available at GitHub [45] with installation and usage instructions and has been deposited to Zenodo [46] under MIT license. Any additional information required to reanalyze the data reported in this paper is available upon request.*

(8) "Especially for hard deconvolution tasks, such as samples with many similar cell types and very scarce cell populations, an increase in deconvolution performance is warranted." I think this sentence needs to be expanded (likely a few more sentences). Currently there is no guide for users to know what is the threshold for defining "very scarse cell populations": is it below 10%? 5%? 1%? Similarly, there are no guidelines for defining "similar cell types". You could mention whether DISSECT worked for X and Y similar cell types in dataset 1, but for A and B similar cell types in dataset 2 it didn't. That would give readers the background on which datasets were used for defining these practical guidelines. Otherwise, right now, users are left blind and need to do full explorations themselves to find these thresholds. That is a lot of work and will turn users away from DISSECT.

That is a great comment and we agree that information about the limits of detection are crucial for end users. DISSECT achieves good performance in distinguishing well defined cell types that include some transcriptional similarity such as CD4 T cells and CD8 T cells (in PBMCs). First we calculated the accuracy of DISSECT on the datasets with ground truth proportions (Rebuttal Fig 2). Only for a few cell types (B cells and NK), there were enough samples (more than 5) for such a comparison. We observed that the rmse remains largely stable with decreasing proportions, however, it is data-dependent. For SDY67, Monaco I and GSE107572, we observed less than 0.04 rmse while for others it was between 0.04 and 0.1

Fig 2. Evaluating errors of DISSECT in estimating cell type proportions for datasets with ground truth proportions. We created 4 subsets from each dataset (indicated as columns). A value of 0.05 in the y-axis indicates that only samples containing a maximum of 5% of a particular cell type (x-axis) were kept. Setting the threshold to less than 0.05 did not result in enough samples for comparisons. Gray colors indicate that not enough samples passed the threshold for comparisons.

In other experiments with similar cell types, DISSECT distinguishes between pancreatic islets - alpha, beta, gamma, and delta cells and shows lower errors than other deconvolution algorithms (Figure S4A) and between tubular epithelial cells such as proximal tubules (PT) and distal convoluted tubules (DCT) (Figure 4D). In our experiment involving granular cell type proportions including CD4 T cell, CD8 T cell, monocytes, and B cell subsets with differing activation states (Table S3), we found that DISSECT estimates had an average correlation of 0.6. However, for some subsets, specifically B NSM from other B cell subsets, we observe low correlation of 0.09 and comparative high root mean squared error (>0.01) with respect to their ground truth proportions. For example, for the B NSM subset, which is present less than 0.01 in all samples, an rmse of 0.02 is a proportional error of 200%.

Based on these findings, we have expanded our original discussion:

DISSECT accurately distinguishes cell types where the transcriptional difference reflects cell subtypes, for instance PBMCs (CD4 T cells and CD8 T cells), pancreas (pancreatic islets), kidney (tubular epithelial cells) and brain (OPC and oligodendrocytes). However, when estimating granular cell type proportions in the Monaco I dataset, error rates exceeded the ground truth proportions (rmse>0.01 for cell subsets present at less than 1%). Therefore, for cell types that make up less than 1% of all cells and cells with very similar gene expression, for instance CD4 T and activated CD4 T cells, deconvolution algorithms should be used with caution.

In addition, we have added a brief Conclusions section as required for a Method paper for Genome Biology.

In conclusion, DISSECT provides a semi-supervised deep learning framework to estimate cell type proportions and per-sample cell type-specific gene expression, is robust across datasets and tissues, and easily applicable to other data modalities. DISSECT delivers state-of-the-art deconvolution performance, as long as cell types are not too closely related and make up more than 1% of all cells.

(9) Note that https://doi.org/10.1101/2023.03.13.532468 evaluated other deconvolution methods that estimate the cell type specific expression levels. They found bMIND to be the best performer. Overall, it seems to me that it would be useful to compare DISSECT against other tools than just TAPE-A.

We have added bMIND together with BayesPrism following the feedback of reviewer 2. The results are included in the updated Table 1. The performance of bMIND was similar to DISSECT in 2 out of 8 comparisons, in mean correlation for DonorC in sample-wise and for PBMC6k in gene-wise correlation. When compared to all other methods, DISSECT still performs best in 6 out of 8 comparisons.

(Table 1)

We have updated the text in the results section to include these results: “We compared the performance of DISSECT with that of TAPE-A, bMIND, and BayesPrism, all of which can infer cell type-specific gene expression per sample.” and “Moreover, DISSECT exhibited an improvement in both sample and gene-wise metrics, exemplifying its advantage.”

(10) The caption for Figure S5 overlaps the page number. This might be out of your control. It also happens with other pages in the supplement.

We have now corrected the figure height and hope that this solves the problem.

Reviewer 2:

In this paper the authors proposed two semi-supervised deep learning models for cell type deconvolution and cell type expression signature estimation, named DISSECT. The MLP model is used for deconvolute cell type fractions. The authors have benchmarked this model on both simulation and real bulk datasets. They also show that their model is possible to be extended to proteomic data and spatial transcriptomic data. Their second model applies a VAE framework to estimate or update cell type specific gene expression. This model is only tested on simulation data. The authors claim that the consistency regularization design in both models can account for the domain shift between the training and target data. Single-cell guided bulk deconvolution is a classic problem, and has been discussed for many years. It is still an interesting topic but may not be as catchy as in past years. While the use of semi- supervised deep learning framework and the application of consistency regularization in DISSECT can be a valuable addition to this field, further efforts are needed to clarify the difference and advancement of this method over the others.

We would like to thank the reviewer for the very detailed and constructive criticisms and suggestions, which have led to both scientific and structural improvements of our work. Especially the feedback to define and visualize the domain shift and show the advantage of consistency regularization has been greatly appreciated.

1. I think the major issue about this manuscript is the how to clarify and elucidate the novelty and advantage of DISSECT over existing methods. For example, if you think the model's ability of domain adaptation is the major advancement, then this should be thoroughly illustrated. First, the definition of "domain" should be clearly elaborated. The words such as domain shift, domain knowledge, domain gap or domain variation may not be familiar to the general audience. Second, the authors seem only hypothesize that consistency regularization can accommodate domain shift between training and target data, but failed to show the evidence. Some visualization of the data can help illustrate existence of domain gap. Comparisons of performance on data with and without domain gap are also necessary.

We thank the reviewer for this excellent constructive criticism. We agree that a detailed introduction to the notion of domain shift, a visualization thereof, and a comparison of algorithmic performance in the presence of explicit domain shift would be very valuable additions to the manuscript. In the revised manuscript, we have now included a detailed introduction to the topic of domain shift in the Background section:

“Domain refers to the statistical distribution of the source of a dataset [10]. Domain shift refers to a change in the statistical distribution of samples, which can be due to covariate shift, the presence of open sets, or both. In gene expression datasets, the covariate shift between real data and simulated datasets occurs due to changes in cell type-specific gene expression and can arise from different dropout rates and tissue conditions, for instance. When domain shifts have purely technical reasons, they are often termed batch effects. CibersortX (CSx) [8] has previously approached the problem of batch effect removal between single cell gene expression datasets [11], using Combat [12] to remove changes in cell type-specific gene expression between a single-cell reference signature matrix and bulk RNAseq data. Open sets may occur when new cell types are encountered during test time, such as the presence of differing cell lineages [13]. Since cells go through different differentiation states, domain shift between real data and simulations may be a combination of both, the covariate shift and presence of open sets.”

Secondly, we have included a figure illustrating domain shift between the simulated and real datasets in Figure S1A of the revised manuscript (rebuttal Fig. 3). We combined the count matrices of simulated data from PBMC8k single cell and bulk data sets. First, we selected 2,000 highly variable genes for each dataset separately and merged the final list to avoid selecting only dataset-specific genes. On the merged list of genes, we computed 50 principal components. The domain shift exhibited in Fig. 3 is visually similar to the batch effect usually encountered in multi-batch single cell RNAseq datasets.

Fig 3. PC (Principal component) embeddings of simulated and real PBMC datasets computed using the union of the top 2000 highly variable genes per dataset.

Further, we implemented an experimental setup to simulate domain shifts. We describe the setup in the results section ‘Evaluation of DISSECT under domain shifts’. Thee setup is described in the methods section ‘Domain shift experimental setup’ with the text ‘The performance of all methods dropped significantly for test sets with domain shifts. However, the drop in performance was much lower for DISSECT than other methods (Figure S6). Further, a clear advantage of semi-supervised learning with consistency regularization is observed in comparison to DISSECT without consistency, especially in terms of rmse.’

Fig 4. Performance of deconvolution algorithms in estimating cell type fractions under domain shift.

2. The necessity of estimation of type specific expression profile from bulk should be explained. If there is enough single-cell data, I don't see why it is needed to make the double step estimation for cell type-specific expression. The result part on Page 19 Line 42 only proves the applicability of the second model. However, it does not convince me of the need for such a model if no real data application is exemplified.

We are greatly sorry that we haven’t sufficiently motivated the gene expression deconvolution task and have taken several steps to improve this in the revised document. We included the following in the Background section:

The estimation of cell type-specific gene expression is a well established and challenging problem in the field. Prior work includes but is not limited to TAPE [5], bMIND [6], BayesPrism [7], and CibersortX (CSx) [8]. The basic aim is to provide cell type-specific gene expression information at a group or sample level. The resultant information allows deep biological insights into cell type-specific gene expression and pathway changes from bulk data.

The reviewer further suggests using single cell data and fraction estimates to reconstruct cell type-specific gene expression. While this might be possible in special cases this approach has two profound limitations. First, a random sampling of single cells would not account for the heterogeneity between bulk samples and cannot capture condition or activation changes in bulk data. Much of the relevant information of the bulk sample would be lost irretrievably. Second, it cannot capture the complete gene expression observed in the bulk sample, as single cell expression suffers from notoriously high levels of drop out. In other words, up to 80% of the gene expression observed in the bulk sample would not be accounted for in the single cell data..

Lastly, we would like to address the last comment of the reviewer, that we prove the applicability of the second model but do not exemplify a real data application. Indeed the results of cell type specific gene expression with DISSECT prove its applicability and advantage over existing state-of-the-art algorithms. We also agree that an application on real data for the evaluation of cell type specific gene expression estimation would be optimal. However, contrary to the setting in the estimation of cell type fractions, where we relied on flow cytometry and well-established biological hypotheses, we could not find similar ground truth in the case of cell type-specific gene expression estimation. We acknowledge this shortcoming in the results, 'While we were able to use ground truth flow cytometry data for the evaluation of cell fractions, no such gold-standard is available for cell type-specific gene expression information.'. In other words, we would love to include real data performance estimates in this manuscript but were and are still unable to obtain such data. A potential solution to this problem would be to generate benchmark datasets with clear gold standard ground truths based on biological insights. We have added the following to the Discussion of the manuscript reflecting this: No gold-standard ground truth exists for quantitative assessment of estimated cell type- specific gene expression between two conditions for real bulk RNA-seq datasets. This is a limitation of the experimental setup presented for cell type-specific gene expression estimation. A potential solution will be to develop biologically valid benchmark datasets that can be evaluated at scale.

Lastly, we would like to stress that this manuscript has a technical focus and is aimed at improving cell type and gene expression deconvolution by using semi-supervised consistency regularization. We do not aim to obtain or present novel biological insights into a specific topic. Suffice it to mention that we have used DISSECT several times to gain deep biological insights, for example in kidney disease, resulting in a novel drug for treatment of human malady (manuscript currently in review at Nature Medicine). However, we are willing to conduct experiments based on the reviewer’s recommendations if deemed essential for or methods manuscript.

3. Regarding the model traning on P.9 L.21, how do you loop over true samples? Or do you just randomly draw a true sample at each step? Also, how batches were generated? Do you divide samples into mini-batches in advance? At each step, you use a true sample and pair with it a simulated pseudo-sample?

Thank you for this question. We have now added a text detailing the mixing procedure in the description of the network under ‘Network architecture and learning procedure’:

*Since the number of simulations is generally larger (in our experiments, set to 1,000 times the number of cell types) than that of real data, we sample real data to create additional bulk samples, Bi., until the size equals that of the simulated data, Bsimi.. This pair of data, together with simulated proportions, Xsimi., is then used to create batches of size 64. For every batch, we generate mixtures according to equation 2.*

4. In the second model, do treat f as known which is a completely trained mapping from model 1? Or can f be also trained in this model?

f is the first model that estimates cell type fractions. We used its outputs without any retraining. We have added the following text under “Estimation of per sample cell type specific gene expression profiles” for clarity:

*f(Bi.)k is the proportion of cell type k in sample i as estimated during cell type fraction estimation and is fixed during the training.*

5. In your simulations studies, how many bulk samples are simulated for each experiment?

We simulated in total 1,000 times the number of cell types in the single cell data. This information is available in the methods section ‘Pre-processing and simulations’.

6. BayesPrism is another methods that perform deconvolution as well as cell type specific expression estimation, which should be compared.

Thank you for bringing BayesPrism to our attention. We have added BayesPrism in our experiments with bulk RNA-seq data. The figures 2 and 3 as well as Figures S2-4 are updated accordingly. Considering the question 2 of reviewer 1, we also included BayesPrism with preselected marker genes and refer to it as BayesPrism-M.

While both versions of BayesPrism perform quite stable across experiments, they are consistently outperformed by DISSECT.

7. In spatial transcriptomics data the number of samples (spots) may approach 5000, how do you adjust the number of steps? There are methods designed with specific for ST data which utilize spatial information, e.g. CARD, SONAR, SD2. How do DISSECT compared to these methods?

In our experiments, there was no need to adjust the number of steps. In both tested Visium datasets, the number of spots (lymph node: 4,035 and mouse brain: 2,695) was considerably lower than the total number of examples the model is trained on. Since we perform training in batches with default value of 64, the model is trained on 64 examples for each batch and in total 320,000 examples over the course of training. However, it should be noted that we only fine-tuned our network for activation functions, learning rate, lambda, and batch size. We fixed the number of steps to 5,000 as found optimal previously in Scaden (Menden et al., 2020). We would like to thank the reviewer for suggesting the comparison to ST data-specific deconvolution algorithms. We have now included CARD and SONAR in our comparisons. As you will find in Figure S8E, on mouse brain data DISSECT outperforms SONAR while performing slightly worse than CARD (a silhouette score difference of 0.02). On lymph node data, however, DISSECT outperforms CARD in the accurate identification of germinal centers and is on par with RCTD and C2L (Figure S9C). By and large the results suggest that DISSECT performs as good as ST data-specific algorithms on the deconvolution task, which is quite a feature given that we haven’t optimized it for spatial deconvolution. Note that SD2 led to poor results and could not identify high neuronal proportions in the cortex (Rebuttal Fig 5). We had to adapt the implementation in SD2 available at https://github.com/leihouyeung/SD2. In particular, we had to change the model import in train.py which by default imports another deconvolution method, DSTG and port the code from tensorflow 1 to tensorflow 2 to work with our computational setup. Since there was no information about package dependencies available, we used trial and error to run the algorithm. This seems to be a known issue (Issue “Dependence of SD2” at https://github.com/leihouyeung/SD2/issues). Considering this, we would prefer to not include the results of SD2.

Fig 5. Spatial deconvolution of a Visium mouse brain slide using SD2. The Figure shows layer 2 (L2) - layer 5 (L5) cortical neuronal subsets colored by deconvolved cell type proportions. The striking absence of clear neuronal layering, as observed for other deconvolution algorithms, clearly demonstrates the suboptimal performance of SD2 in our hands.

8. The evaluation in section "Estimation of cell type-specific gene expression" should be clarified. It is not clear how sample-wise and gene-wise correlations are calculated.

We have added the following text to clarify this:

To evaluate the performance of DISSECT and other methods, we compared the true and estimated gene expression profiles of each cell type for each simulated sample (sample-wise) and for each gene (gene-wise) using Spearman correlation. These sets of results were aggregated across cell types and averaged.

9. It is not clear how ablation study was performed (P.14 L.41)

We understand that there was not enough information and we greatly appreciate this comment. We have modified the text to provide a clear description:

To validate that the performance improvement in DISSECT is due to the semi-supervised learning and consistency loss, we performed an ablation study on data SDY67 by successively and cumulatively removing components of the algorithm and testing it again. The following components were removed successively: consistency regularization, KL Divergence loss (mean squared error instead), and the nonlinear activation function (identity function instead).

The ablation results are shown in Table S5.

Minor comments:

10. Can you elaborate or at least briefly show how the grid search was performed in choosing the optimal activation function.

We fine tuned the network for activation functions, learning rate, and batch size using randomized search with hyperopt (http://hyperopt.github.io/hyperopt/) with the root mean squared error as the objective function. The following grids were used for the optimization: activations = [Linear, ReLU, ReLU6, Swish], learning_rate = [5e-3, 1e-3, 5e-4, 1e-4, 5e-5, 1e- 5], λ1 = [0,1,5,10,15] with or without a scheduled change at every 2,000 steps and batch sizes = [32, 64, 128, 256] with 50 iterations on Ascites bulk dataset as used in Scaden (Menden et al., 2020). Other hyperparameters were fixed to the default hyperparameters of Scaden. We have added a section detailing this under ‘Hyperparameters and fine-tuning’ in the Methods.

11. Do you have to re-tune the hyper-parameters such as \lambda_1, \lambda_2, \beta^VAE and the learning rate each time when applying DISSECT to a new dataset?

We used the default settings in all experiments to make comparisons fair for all algorithms.

The following changes were made for specific modalities:

Spatial transcriptomics: We adjusted the simulation procedure and used the output of the penultimate neuronal layer.

Proteomics: We adjusted the mixing procedure during training

Both of these changes have been described in the Methods under ‘Pre-processing and simulations’. The optimal parameters along with the details on fine tuning are now added to the section ‘Hyperparameters and fine-tuning’.

12. In the sentence on P.5 L.41, "… consistency-based regularization penalizing the non- linearity of mixtures of real and simulated samples…", can you explain what is the non-linearity of mixtures of real and simulated samples?

By non-linearity of mixtures of real and simulated samples, we mean a function f that satisfies f(Bmixi.) != 𝐀f(Breali.) + (1-𝐀)f(Breali.). We have added the following statement at the end of the respective paragraph to clarify this and thank the reviewer for this comment:

Non-linearity of mixtures of real and simulated samples refers to the violation of Equation 4, defined later, for estimated Xi., Xsimi. and Xmixi. using mapping f.

13. The author proposed two models but only gave one name, DISSECT. I would be more convenient if each model is assigned with a sub-name.

We appreciate the feedback to improve the accessibility of DISSECT. We are a bit hesitant to change the name as both algorithms use the same principle, semi supervised consistency regularization. In case the reviewer and editor see a strong benefit in changing the names to DISSECT-c and DISSECT-g, for instance, we would obviously heed your advice.

14. P.10 L.14, CSx should be presented in full name for the first appearance in the MS.

Thank you for noticing this. We have corrected this in the revised manuscript

15. There are some typos： P.10 L.41 should it be "-" instead of "+" for the last term? P.11 L.48 … two evaluate…-> to evaluate P.22 L.37 there are 2 "y" in eqn7

We have corrected the typos accordingly

**2^nd^ round**

**Reviewer 1**

Summary:

Earlier this year (likely after the DISSECT updates from 3 months ago https://github.com/imsb-uke/DISSECT/commits/main/) someone in my research group tried to test DISSECT their main feedback was that the documentation was too sparse. This is why I'm putting more of an emphasis on the software documentation than the actual paper. Overall, I do think the paper illustrates why DISSECT would be interesting to try out if you have a dataset you want to deconvolute. But if readers then attempt to become users and run into a software that is lacking in documentation, you might never even hear from them at https://github.com/imsb-uke/DISSECT/issues if they feel that they just have too many questions to even get started in comparison to competing software tools. Given that you are describing new software, I do think that it would be good to improve the documentation to help drive up citations of your good manuscript.

Overall, I do appreciate the improvements that were made to the software documentation, creating https://imsb-uke.github.io/DISSECT/, making the analysis code publicly available, and the responses to my previous comments. The manuscript edits are most welcome. I can tell that a lot of great work was put into this revision.


Main comments:

I do appreciate the improvements to https://github.com/imsb-uke/DISSECT/tree/main?tab=readme-ov-file#installing, the tutorials, and documentation such as the comments at https://github.com/imsb-uke/DISSECT/blob/main/dissect/configs/config.py. Though some of the terms are not documented such as https://github.com/imsb-uke/DISSECT/blob/2846d2b9fc69550bdafab02e87c716fa9b41bc7e/dissect/configs/config.py#L41 and https://github.com/imsb-uke/DISSECT/blob/2846d2b9fc69550bdafab02e87c716fa9b41bc7e/dissect/configs/config.py#L67. Other ones are confusing like https://github.com/imsb-uke/DISSECT/blob/2846d2b9fc69550bdafab02e87c716fa9b41bc7e/dissect/configs/config.py#L10-L11 which seems to have a contradiction between the default value of "None" and the documented default value of 500. Or does "None" actually mean 500 in this case? I also don't understand what the acronyms "srm" or "rrm" stand for, even with the comment describing them at https://github.com/imsb-uke/DISSECT/blob/2846d2b9fc69550bdafab02e87c716fa9b41bc7e/dissect/configs/config.py#L42. Also, maybe I missed it, but it doesn't seem like a user can see the definition of the variables (something like a "DISSECT --help" command). You can only see the output from reading the json config file with no comments like at https://github.com/imsb-uke/DISSECT/blob/main/tutorials/tutorial.ipynb under "Import DISSECT module".

Similar to the above point, there seems to be no documentation describing the actual input data formats. Sure, there are comments like https://github.com/imsb-uke/DISSECT/blob/2846d2b9fc69550bdafab02e87c716fa9b41bc7e/dissect/configs/config.py#L33 but do you have to use rownames for the gene IDs? Should they be ENSEMBL or GENCODE or SYMBOLs (names)? One could compare against the example data that is provided, but it would be best if there was more detailed documentation on the input data formats. This is one of the main comments from the person in my research group who tried using DISSECT.

At https://github.com/imsb-uke/DISSECT/blob/main/tutorials/tutorial.ipynb under "Estimation of cell type specific gene expression" it seems like it would be nice to see how one can visualize the estimated cell type specific expression. Maybe some boxplots / violin plots for a given cell type marker gene across the bulk samples, or a scatterplot comparing the raw bulk expression against a specific cell type expression for a given gene. It currently basically ends with: we get a Scanpy object and you can now use Scanpy functions as you would normally. The PCA and highly variable genes sub-sections have no comments besides codes and plots. Overall, I prefer tutorials to be a bit more verbose to fully guide users. I recommend asking someone else in your group who hasn't worked so closely with DISSECT to try to install the software and follow the tutorial. That person will likely have several questions that will help the developers improve the documentation. That's because as a developer it can be easy to forget the background that users need to understand in detail how to use the software.

The spatial tutorial at https://github.com/imsb-uke/DISSECT/blob/main/tutorials/tutorial_spatial.ipynb shows a PC plot with overlapping labels that cannot be read. I'm also confused under "Import DISSECT module" as to why it refers to bulk data. This makes me think that the comments were copy-pasted from the main tutorial and not updated for spatial data. I was particularly curious if one had to change the config file in any way for applying DISSECT to spatial data.


As far as I can tell, there are no unit tests or any type of continuous integration tests for DISSECT. I would encourage you to work on these post-publication of your manuscript, as they will help both your users as well as yourselves to detect bugs early and address them. Some great advice I've gotten in the past is to use user bug reports to build new unit tests. Of course, only if the data is small enough or the issue can be replicated with a small dataset.


Minor comments:

* Page 31, starting on line 22: "Any additional information required to reanalyze the data reported in this paper is available upon request.". Related to this, I do see the code at https://github.com/imsb-uke/deconvolution/tree/main. I would encourage you to deposit it on Zenodo (or Figshare or some other DOI-granting location for code) just like you did for https://github.com/imsb-uke/DISSECT.
* "The code has been deposited to Zenodo and is available with the following link and DOI: https://zenodo.org/doi/10.5281/zenodo.10570403.". I would just add the Zenodo badge at https://github.com/imsb-uke/DISSECT.
* I made the easy to make mistake of jumping from https://github.com/imsb-uke/DISSECT/tree/main?tab=readme-ov-file to https://github.com/imsb-uke/DISSECT/tree/main/tutorials instead of reading all the information at https://github.com/imsb-uke/DISSECT/tree/main?tab=readme-ov-file#tutorials. I was then left wondering what each file contained. I recommend moving some of the information from https://github.com/imsb-uke/DISSECT/tree/main?tab=readme-ov-file#tutorials to a README.md at https://github.com/imsb-uke/DISSECT/tree/main/tutorials such that the documentation about each tutorial is next to the actual file. Alternatively, you could use direct links to each tutorial file from the main repository README.md file instead of linking to the Tutorials directory.
* Page 2, line 52: missing space in "data.Domain"
* Page 12, line 27: datsaets -> datasets.
* Page 19, line 18: missing space in "information.For"
* Page 30, line 40: "10x genomics" -> "10x Genomics" is how they spell their company name. You did spell it correctly elsewhere, like in page 42 line 41.
* Update any preprint references if they have been published. I see several aRxiv and bioRxiv citations.
* Figure S6: "Actiated 2" -> "Activated 2"
* Figure S8: you could show it larger and have the caption on another page if you need the space. I cannot read the text on panel C unless I zoom in at 200%. Similarly with some text in panel D.

**Reviewer 2**

The authors have made great progress in this revision, and they have addressed my concerns.

In your response to my Q3 and in P.9 L.26 of the revision, "we sample real data to create additional bulk samples, B_i.". Do you mean "B^mix_i." here?

I also have an addition curiosity, if I iteratively run DISSECT-c and DISSECT-g, will I get better and more fine-tuned estimates of cell type fraction and type specific expression profile?

**Authors’ response**

We made changes to the code documentation as required by the reviewer 1.

Typos mentioned by the reviewer 1 are corrected.

We added a statement in the discussion satisfying the question of the reviewer 2
